# Supplementary material for: Segregation and polarization in urban areas
Source: R Soc Open Sci. 2019 Oct 23;6(10):190573. doi: 10.1098/rsos.190573 (PMC6837204; doi:10.1098/rsos.190573)
Supplement: Supplementary Material [file rsos190573supp1.pdf]

Segregation and Polarization in Urban Areas

Alfredo J. Morales[1,2], Xiaowen Dong[2,3], Yaneer Bar-Yam[1] and Alex ‘Sandy’ Pentland[2]

[1]New England Complex Systems Institute. [2]MIT Media Lab. [3]University of Oxford.

Extended Data

S1 Data sources

The Twitter data sets were created using the Stream Application Programming Interface (API). Twitter is an online social network whose users share “micro-blog” posts from smartphones and other personal computers. In total we collected over 87 million geolocated tweets from over 2.8 million users between August 2013 and August 2014. Geolocated tweets provide a precise location of the individuals that post messages, and represent around 3% of the overall Twitter stream [1]. Table S1 shows the total number of tweets collected per city.

City	Tweets	Users
Chicago	11,035,979	356,907
Dallas	7,484,473	230,989
Detroit	4,678,721	127,643
Istanbul	26,086,944	667,288
New York City	21,459,704	753,251
Los Angeles	12,376,883	507,736
Philadelphia	4,313,839	179,511

Table S1: Twitter data sets. Number of geolocated tweets and users analyzed by city.

In order to further assess how representative our Twitter sample is, we compared the number of Twitter users we identified living in each neighborhood with the neighborhood population taken from Census data. Home locations are inferred through individual patterns of nighttime Twitter activity. The results are presented in Table S2. The correlations are all significant and particularly high for four cities.

City	r	p-value
Chicago	0.56	5.56E-183
Dallas	0.61	9.33E-138
Detroit	0.63	4.29E-146
Istanbul	0.57	2.41E-58
Los Angeles	0.32	2.05E-67
New York City	0.40	2.27E-79
Philadelphia	0.39	1.47E-54

Table S2: Pearson correlation (r) of Twitter and neighborhood population. The p-value shows the probability that an uncorrelated system produces a correlation of at least r.

The credit card purchase data set was provided by a major financial institution in Istanbul. Records correspond to purchases with credit cards and include the customer (user) and store identifier. Store and user home locations are provided as meta data, together with users' income. The data has been properly annonymized following the current Turkish privacy laws. We analyzed a total of 2.4 million records of individual credit card purchases in a three-month period, made by 85 thousand individuals at 54 thousand stores.

In Table S3 we show the ranges of the income quantiles (Q) used to create Figures 1 and 2. The table elements show the upper limit of the specific quantile in each city. The lower limit is given in the previous quantile element.

Q	Chicago	Dallas	Detroit	New York City	Los Angeles	Istanbul	Philadelphia
0	27,143	30,413	21,250	26,566	31,014	21,500	27,647
1	37,423	37,865	28,222	35,096	37,260	23,263	39,234
2	44,773	43,477	35,932	41,615	43,585	24,638	49,761
3	51,667	49,592	44,337	47,477	51,103	25,918	57,099
4	58,750	56,442	50,506	53,882	58,125	27,669	63,952
5	659,78	64,355	57,698	60,439	66,725	29,926	71,623
6	74,778	73,125	64,972	67,958	76,546	32,686	81,178
7	86,144	89,119	75,634	78,652	88,575	37,182	92,528
8	103,606	109,852	93,639	93,393	106,069	44,601	108,750
9	227,708	250,001	177,829	250,000	250,001	80,822	202,857

Table S3: Income range of quantiles (Q) per city. Income is shown in USD and it is annually based.

S2 Interaction matrices

In order to study patterns of segregation, we built networks of social interactions among neighborhoods according to three types of data: shopping, mobility and communication. Neighborhoods represent official sub-urban administrative units defined by national authorities: *mahalle* in the case of Istanbul and *Census tract* in the case of American cities.

The shopping network was created by linking users' home neighborhoods to the neighborhoods where they shop, as determined from credit card transaction data sets. The mobility network was obtained by first analyzing individual patterns of nighttime Twitter activity to infer their home locations, and linking users' home districts to the districts they visit and tweet from. Similarly, the communications network was created by linking the home locations of users mentioning each other in their tweets.

To obtain the interaction matrices in 1 and 2, we first normalize each element of these raw interaction matrices by dividing it by the sum of the corresponding column, and then subtract from it the expected value for a uniform probability of interaction ($p_u = 1/q$). Patterns of segregation are noticeable in the red diagonal of the matrices.

S2.1 Statistical test

We measured the statistical significance of segregation patterns in the interaction matrices shown in Figures 1 and 2 by applying a two-sided Kolmogorov-Smirnov test [2] to samples of social interactions. The null hypothesis is that the interaction origins conditioned on their target follow a uniform probability distribution. The KS statistical test estimates the probability that two samples follow the same distribution. The first sample is drawn from the data by (1) selecting a target quantile Q to analyze and (2) sampling the source of N interactions. The second sample is artificially generated by creating a set of N interactions whose target is Q and whose origins equally represent all quantiles. A total of 10 sets of samples are created for each network (one per target quantile); therefore, 10 tests are applied in each case. The results of all tests across all networks and cities are summarized in Table S4, using samples of $N = 1000$ and $N = 5000$ interactions. The results show that (1) the segregation patterns are statistically significant, (2) the interactions of the middle quantiles are consistently more similar to a uniform probability distribution than other quantiles, and (3) the highest and lowest quantiles are the most segregated groups.

S2.2 Balanced sample

In order to assess the robustness of the segregation patterns shown in the interaction matrices (Figures 1 and 2), we created samples that are balanced following the relative number of people by neighborhood according to Census data. This method under-samples neighborhoods that are over-represented and over-samples those that are under-represented. The size of a random sample

	$N = 1000$			$N = 5000$		
Q	$p < 0.05$	$p < 0.01$	$p < 0.001$	$p < 0.05$	$p < 0.01$	$p < 0.001$
0	93%	86 %	86%	100%	100%	100%
1	100%	93%	93%	100%	100%	100%
2	93%	73%	66%	100%	100%	100%
3	73%	46%	40%	93 %	93%	86 %
4	66%	46%	40%	100%	86%	80 %
5	73%	73%	53%	100%	100%	93 %
6	93%	86%	86%	100%	100%	100%
7	100%	100%	93%	100%	100%	100 %
8	100%	100%	100%	100%	100%	100%
9	100%	100%	100%	100%	100%	100%
All	89%	80%	76%	99%	98 %	96%

Table S4: Results of the Kolmogorov-Smirnov statistical test applied to samples of social interactions. The null hypothesis is that the interaction origins conditioned on their target (Q) follow a uniform probability distribution. A total of 10 tests are applied to each network (one per target quantile Q). There are 15 networks (two for each American city and three for Istanbul). Therefore, each row represents a total of 15 tests. The last row includes all 150 tests. Table values indicate the percentage of tests at each level of statistical significance grouped by Q across all networks and cities, for samples of size $N = 1000$ and $N = 5000$ interactions.

is proportional to the neighborhood population, following the equation:

$$s_i = \frac{c_i}{\sum_j c_j} \sum_k t_k \tag{1}$$

where s_i is the sample size of neighborhood i, c_i is the population (according to Census data) of neighborhood i, and t_i is the Twitter population of neighborhood i. If $s_i > t_i$ we pick a random sample of unique users in neighborhood i not greater than t_i.

In Figure S1 we present the interaction matrices using balanced samples for all cities. The matrices have been normalized and colored as in Figures 1 and 2 (see Section S2). Assuming the uniform distribution as non-segregated and using it as reference (white), values above it (red) show a higher interaction preference and values below it (blue) show a lower interaction preference among the population of income quantiles. The patterns of segregation are just as noticeable as in previous figures. Matrices show a clear red diagonal indicating strong preferences of income quantiles to interact with their own class. In some cases the diagonal structure is even clearer than in previous figures. In New York City, a balanced sample shows that lower income areas visit richer areas (Manhattan) and not the opposite.

S3 Dimensionality reduction

In order to study the structure of both hashtag and mobility spaces, we applied dimensionality reduction to matrices in which rows represent neighborhoods and columns indicate either hashtags posted in those neighborhoods or users who tweet from those locations. The former is an indicator of online conversations and the latter is an indicator of the space of human mobility. Before applying dimensionality reduction, we applied a normalization transformation to the matrices weighting terms according to their frequency of appearance [3]. We then applied PCA analysis and topic modeling to these matrices.

S3.1 PCA analysis

Similarly to Figure 4, in Figure S2 we show the results of the PCA analysis of the hashtag space versus the mobility space, with respect to income for Chicago, Dallas, Detroit, Los Angeles and Philadelphia. Dots represent neighborhoods, and colors indicate income. The patterns in these plots manifest that rich and poor neighborhoods are located in separate data spaces.

The geographical distributions of the hashtag usage and human mobility PCA are shown from Figures S3 to S16. There is one figure for each city and feature space (hashtag and mobility). Panels respectively show the geographical structure of each of the top 20 principal components. The correlation with income of each component is annotated in each panel. The figures show that the main components have clearly defined spatial structures and that one of them (mainly the first or sometimes the second or third) has a high correlation with neighborhood income (also shown in Figures 22 and 23). Higher order components (above the 5th component) explain less variance and show low correlation with income and no clearly defined spatial structure. In the case of human mobility, spatial correlations persist at higher order components, in comparison with the hashtag which becomes random rapidly (see Figures S3 to S16).

In Figure S17 we present the correlation of the top 20 components with income for both hashtag (solid blue line) and mobility (solid red line) matrices in Istanbul and New York City. Similar results for other cities are presented in Figure S18. In all cities one main component (or two at most) is highly correlated with income. In the case of Istanbul, this component is the first one. In other cases, it is the second or third, but never a higher order component. In the case of Dallas, it is the 4th component in the human mobility PCA. Higher order components do not have high correlations with income. In the case of Istanbul, we also present the correlation with political preferences, measured as the percentage of votes to the ruling party by neighborhood (dashed lines Figure S17). The correlation of the first component with political preferences is lower than the correlation with income.

In order to determine the significance of the correlations, we randomly shuffle the hashtag and mobility vectors among neighborhoods, i.e. randomly assign the hashtag or mobility vector of one neighborhood to another, and apply PCA to the randomized space. We repeat the PCA

analysis on the randomized space and calculate the correlation of each component with income. We repeat this experiment 100 times and analyze the statistical behavior of the correlations. The results are presented in Figure S17 for Istanbul and New York City, and in Figure S18 for the rest of cities. The green and yellow lines show the correlation of the components after randomizing hashtag usage and mobility vectors among neighborhoods. Error bars show standard deviation. The correlation of main components with income is clearly significant for all cities ($p < 0.001$). The p-values of the correlation of each component with income are shown in Figure S19. Higher order components are not always significant. Moreover, randomizing the internal structure of hashtag and mobility vectors does not yield any correlation with income in any component.

S3.2 Topic modeling

In order to analyze the significance of topics' correlation with neighborhood income, we applied the topic model to randomized hashtag spaces. We randomized the hashtag space by shuffling the hashtag vectors across neighborhoods i.e. randomly assign the hashtag vector of one neighborhood to another. For each randomization we calculate 20 topics and measure the correlation of each topic with neighborhood income. In Figure S20 we present the distribution of correlations of neighborhood income with the topics obtained from the randomized hashtag space. The black curve shows the distribution of income correlations across all topics, the red curve shows the distribution of minimum correlations and the blue curve shows the distribution of maximum correlations. The vertical blue and red dashed lines show the correlations of the topics used in Figures 5 and 6.

In Table S5 we present the top 10 hashtags related to topics positively and negatively correlated with income in the city of Istanbul. In this city, richer areas are predominantly using hashtags in English that are related to lifestyle. On the other side, in poorer areas, people use hashtags in Turkish related to religion, politics and TV shows.

In Table S6 we summarize the hashtags from American cities by showing the top 30 hashtags that are common in topics positively and negatively correlated with income across all cities. While there are similarities in this case, richer areas continue to predominantly talk about lifestyle, while poorer areas seem to be more interested in horoscopes. The extensive list of hashtags per topic and city in American cites is presented from Tables S7 to S12.

S4 Cultural Distance

Previous studies use the accuracy of prediction tasks in order to measure the distance or difference between cultural features [4]. They assume that low accuracy represents high differentiation among groups and high accuracy represents less differentiation. Using the same methodology, we define differences among income groups via the accuracy of predicting mobility patterns and

Topic-1 ($r > 0$)	Topic-2 ($r < 0$)
love, night, sunday, food, summer, photooftheday, breakfast, yummy, huzur, party, fashion, sea, nofilter, istanbul	np, GazzedeKatliamVar, offline, Soma, FF, GazzeSiyonizmeMezarOlacak, beyazshow, SomadakiKardeslerimize-YardimEtALLAHIM, GazaUnderAttack, medcezir, nw, 3Adam, takipedenitakipederim, siirsokakta

Table S5: Top 10 hashtags from topics positively ($r > 0$) and negatively ($r < 0$) correlated with income in Istanbul.

Topic-1 ($r > 0$)	Topic-2 ($r < 0$)
tbt, usa, love, family, blessed, life, selfie, thestruggle, sorrynotsorry, smh, tired, work, awesome, awkward, 1, help, winning, bye, truth, esurancesave30, fml, 2, wcw, why, mcm, goodnight, college, oomf, wtf, worldcup	wcw, tbt, oomf, np, pisces, mcm, turnup, leo, 1, rt, goodnight, cancer, rip, blessed, thestruggle, love, family, scandal, aries, sorrynotsorry, smh, salute, raw, retweet, wce, libra, 2, thiscouldbeusbutyouplayin, lhhatl, facts

Table S6: Top 30 common hashtags from topics positively ($r > 0$) and negatively ($r < 0$) correlated with income across all American cities.

hashtag usage.

Users are characterized by two vectors that respectively represent their mobility and hashtags posted. The non-zero elements of the mobility vector represent city neighborhoods the individual visits and tweets from. The non-zero elements of the hashtag vector indicate the hashtags individuals post. The vectors are transformed using TF-IDF [3]. This transformation is often used to classify documents and highlights local information as opposed to globally used terms.

We predict the individual income quantile using an MLP classifier independently applied to both the mobility and hashtag feature space. For this purpose, we randomly divide the sample in a training set made of 75% of individuals and a test set made of 25% of them. We create multiple sample sets in order to analyze the performance of the predictor as a random variable. Bootstrapping the performance enables more robust understanding of the prediction quality.

The bootstrap of the prediction accuracy is shown in Figure S21 for the city of Istanbul. Both hashtag (orange) and mobility (blue) are significantly higher than the probability of a random guess given the number of possible categories (dashed line). Therefore people of similar quantiles also have similar behaviors. Mobility patterns are a better predictor of income quantile than hashtags

used. The hashtag space is larger and more diverse. Therefore, aggregation is necessary to observe patterns of collective behaviors of hashtag usage, as those shown in Section S3.

S5 Distributions

In Figure S22 we present the cumulative density function (CDF) of pairwise neighborhood hashtag usage similarity for Istanbul. We measure hashtag usage similarity by the cosine distance of the neighborhoods' hashtag vectors. We grouped neighborhoods by income in 30 quantiles, representing each curve. The color of each curve indicates the normalized median income of each group (from red to blue) and the black dashed curve shows the global distribution. Wealthier neighborhoods (blue) present a significantly higher hashtag usage similarity relative to the global distribution, indicating a more coherent behavior. Meanwhile, poorer neighborhoods show a significantly lower behavior relative to the global distribution, indicating more dispersed conversations.

S6 Aggregation

We analyze the relationship between mutual exposure (measured via inter-neighborhood mobility) and homogenization (measured via the cosine distance between neighborhood hashtag vectors) by means of aggregation and correlation. We aggregate neighborhoods in 10 groups either by income or random association (similar to Figure 7). The interactions within and across groups are represented as matrices. The matrices are normalized as described in Section S2.

As in Figure 7, the left panels of Figure S23 show the mobility and hashtag similarity matrices for Chicago, Dallas, Detroit, Los Angeles and Philadelphia aggregated by income, along with scatter plots showing the respective element-wise correlation of the two matrices. Analogously, the right panels of Figure S23, show the corresponding randomized versions of the matrices and respective scatter plots. A strong correlation between the elements from the mobility and hashtag similarity matrices is manifest in the scatter plots (similar to the results presented in Figure 7).

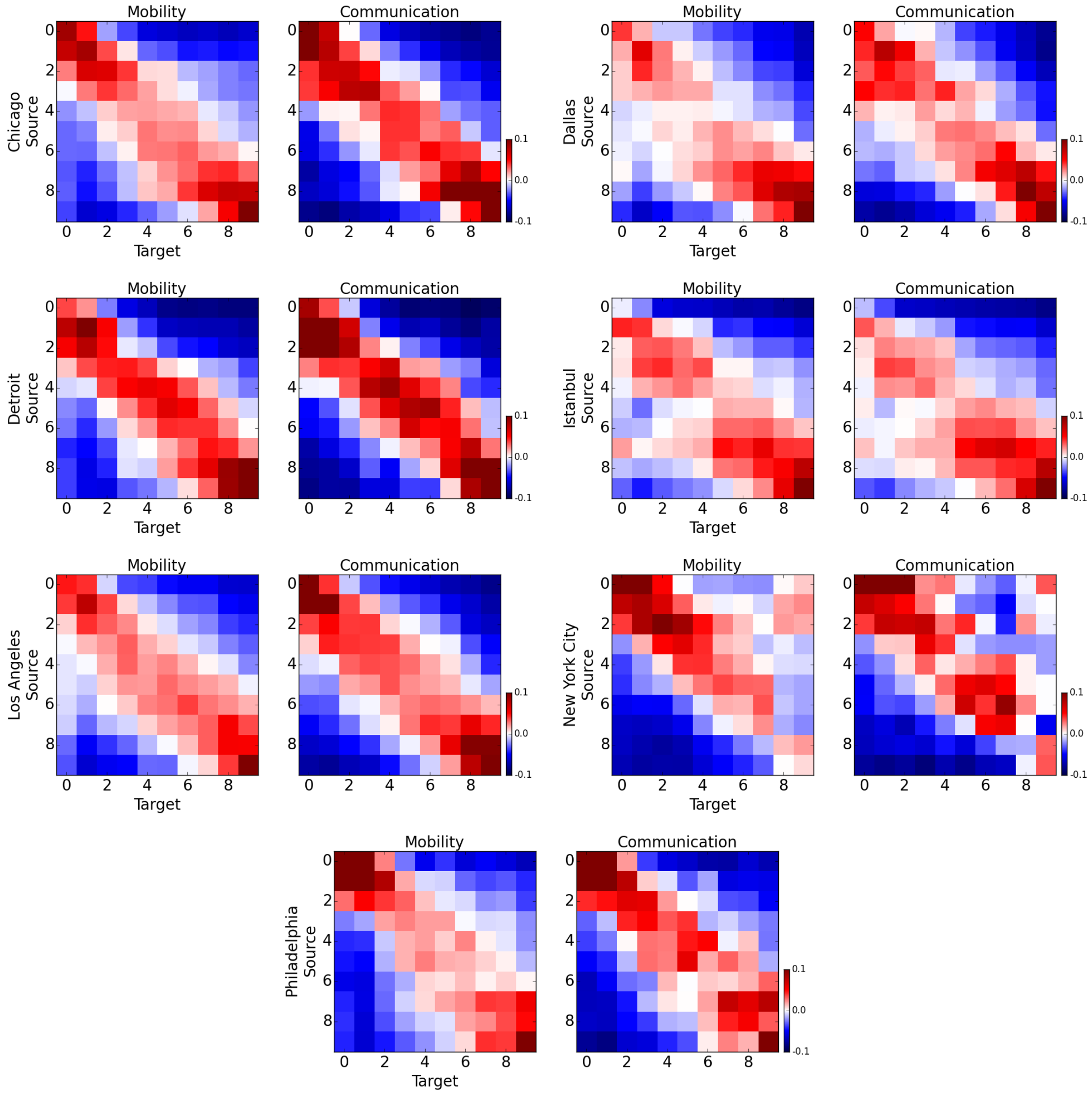

Figure S1: Interaction matrices by type of activity for all cities using balanced samples. Matrices show normalized sum of interactions between each pair of neighborhoods according to mobility (left) and communication (right) on Twitter. Neighborhoods are aggregated into $q = 10$ income quantiles represented in the axes. Blue regions represent the pairs of income quantiles where probability of interaction is below the expected value of a uniform distribution $p_u = 1/q$, and red ones above. The samples are weighted proportionally to the Census population.

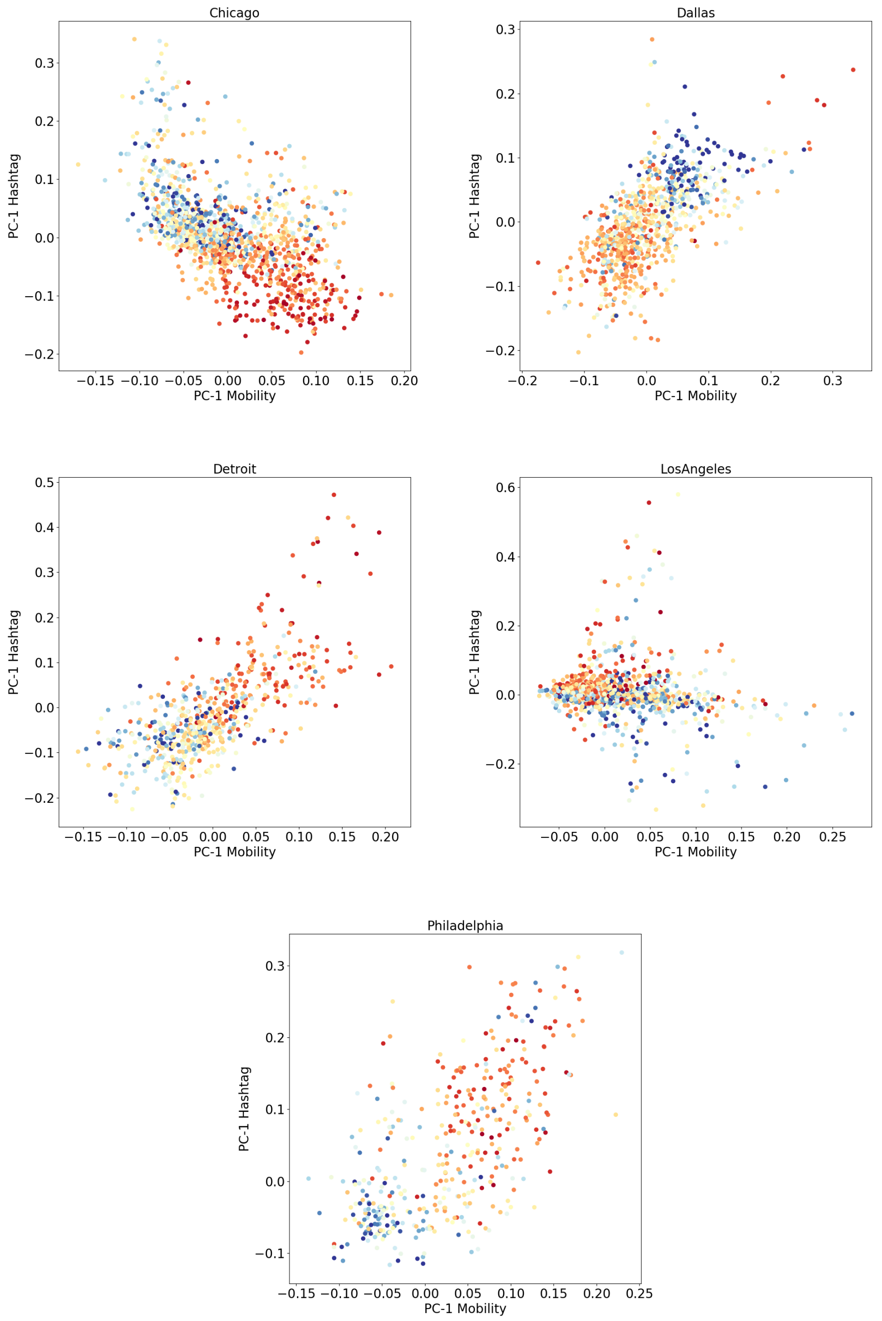

Figure S2: Principal component analysis (PCA) of hashtag usage (y-axis) and human mobility pattern (x-axis) for Chicago, Dallas, Detroit, Los Angeles and Philadelphia. Dots represent neighborhoods, and colors indicate the median income (increasing from red to blue). Each axis represents the projection of the neighborhood data onto one main component correlated with income (see Table 2 in the main text).

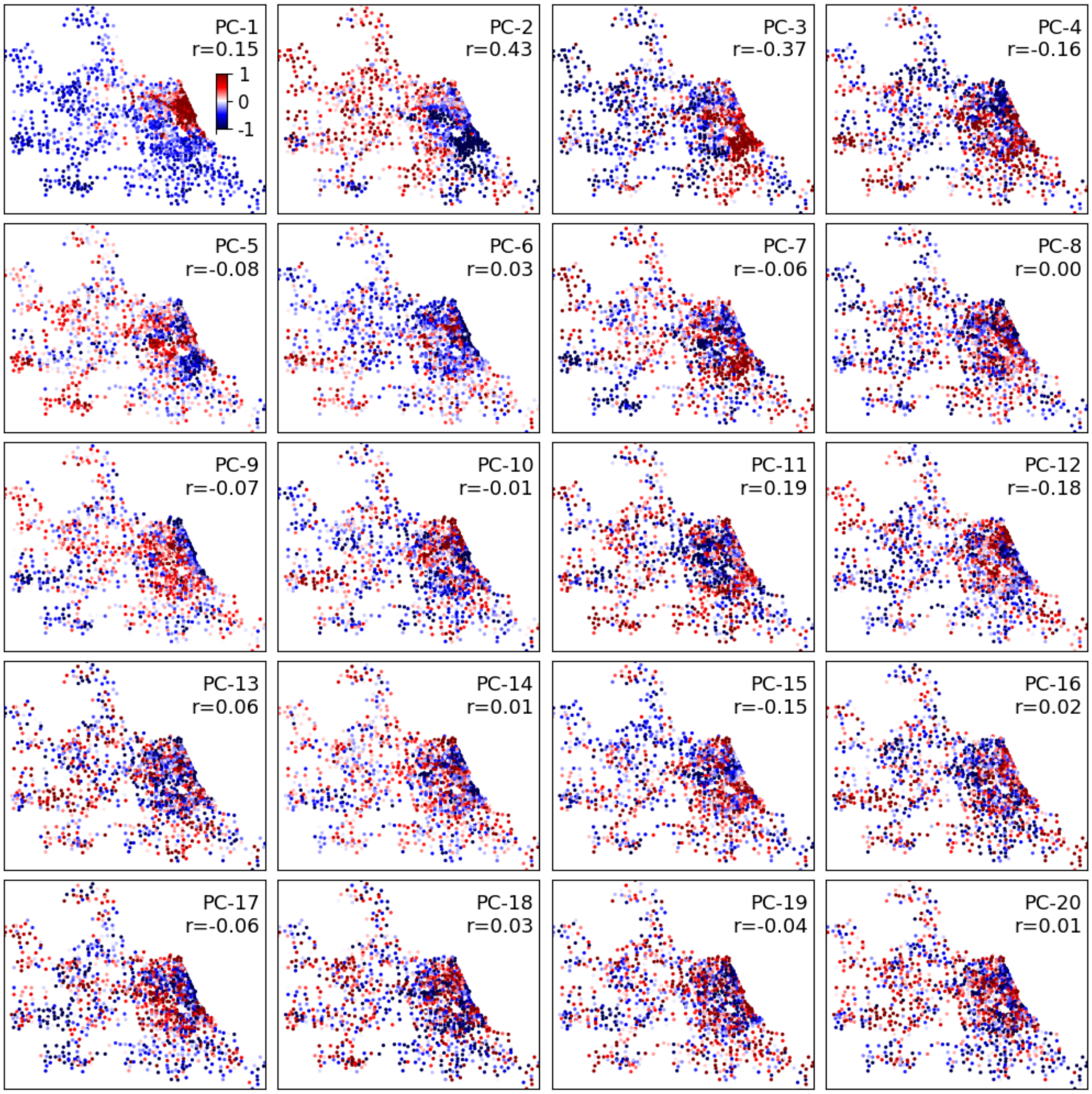

Figure S3: Spatial structure of the 20 principal components of hashtag usage in Chicago. The correlation with neighborhood income r is shown in each panel.

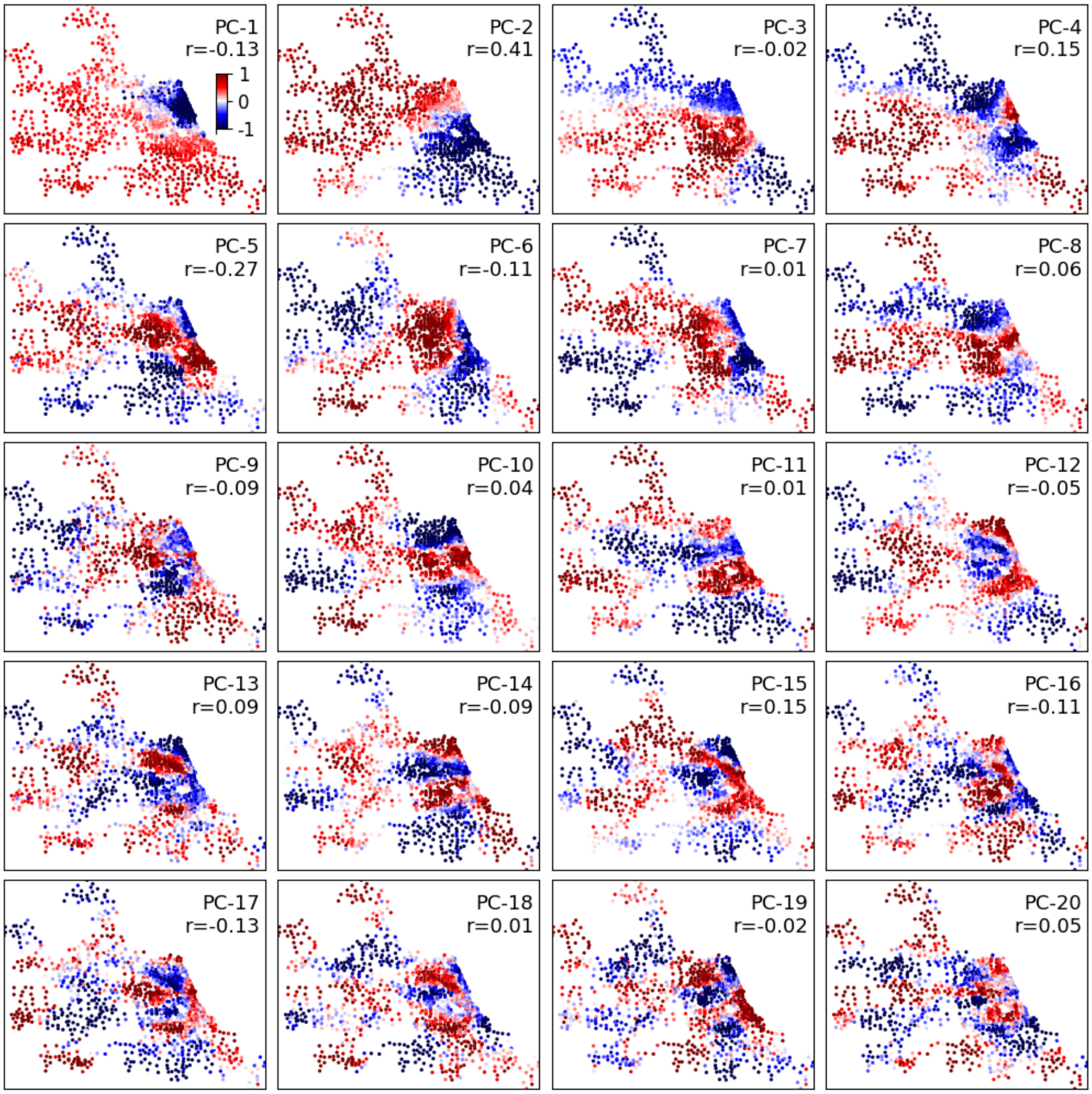

Figure S4: Spatial structure of the 20 principal components of human mobility patterns in Chicago. The correlation with neighborhood income r is shown in each panel.

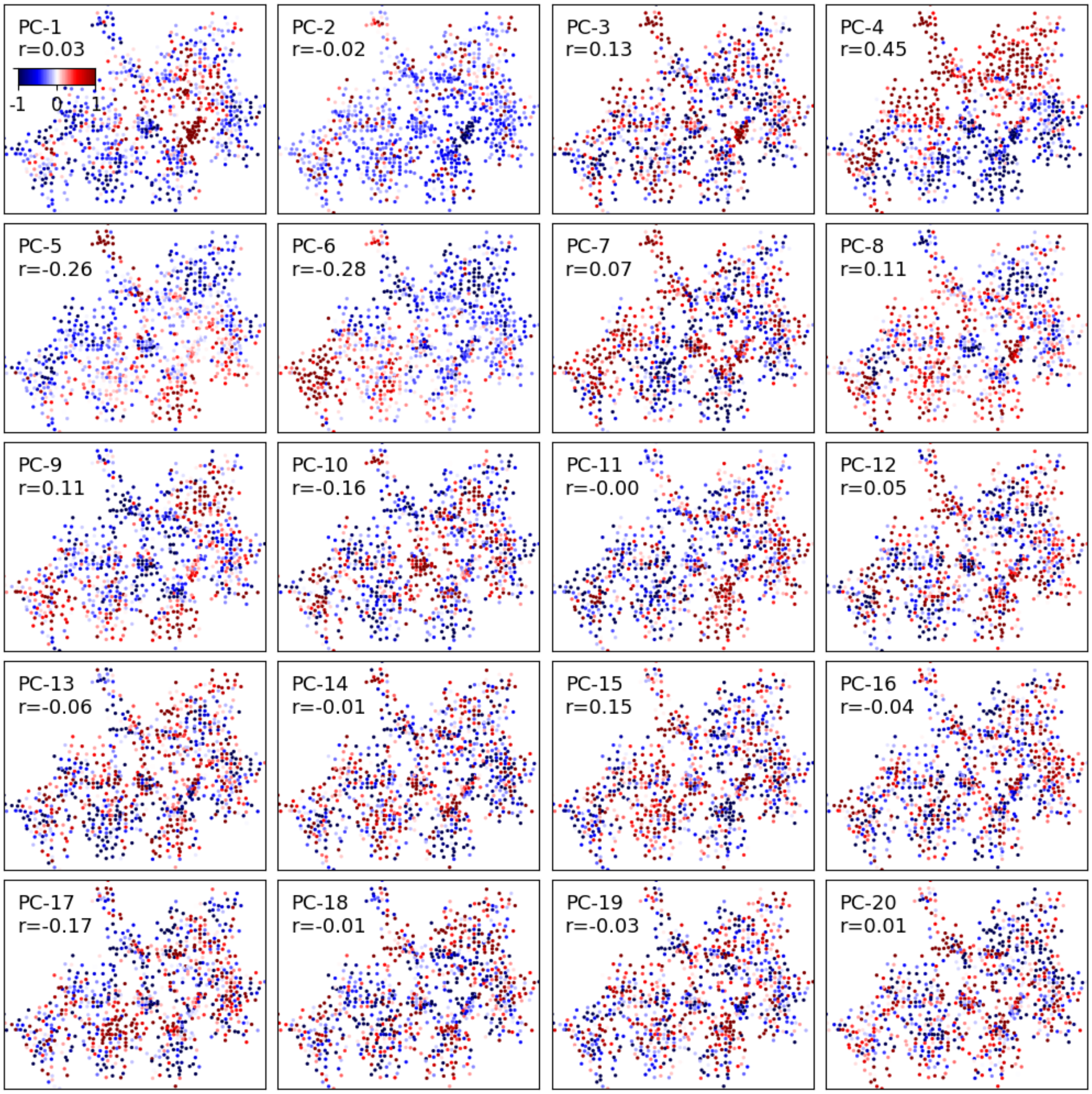

Figure S5: Spatial structure of the 20 principal components of hashtag usage in Dallas. The correlation with neighborhood income r is shown in each panel.

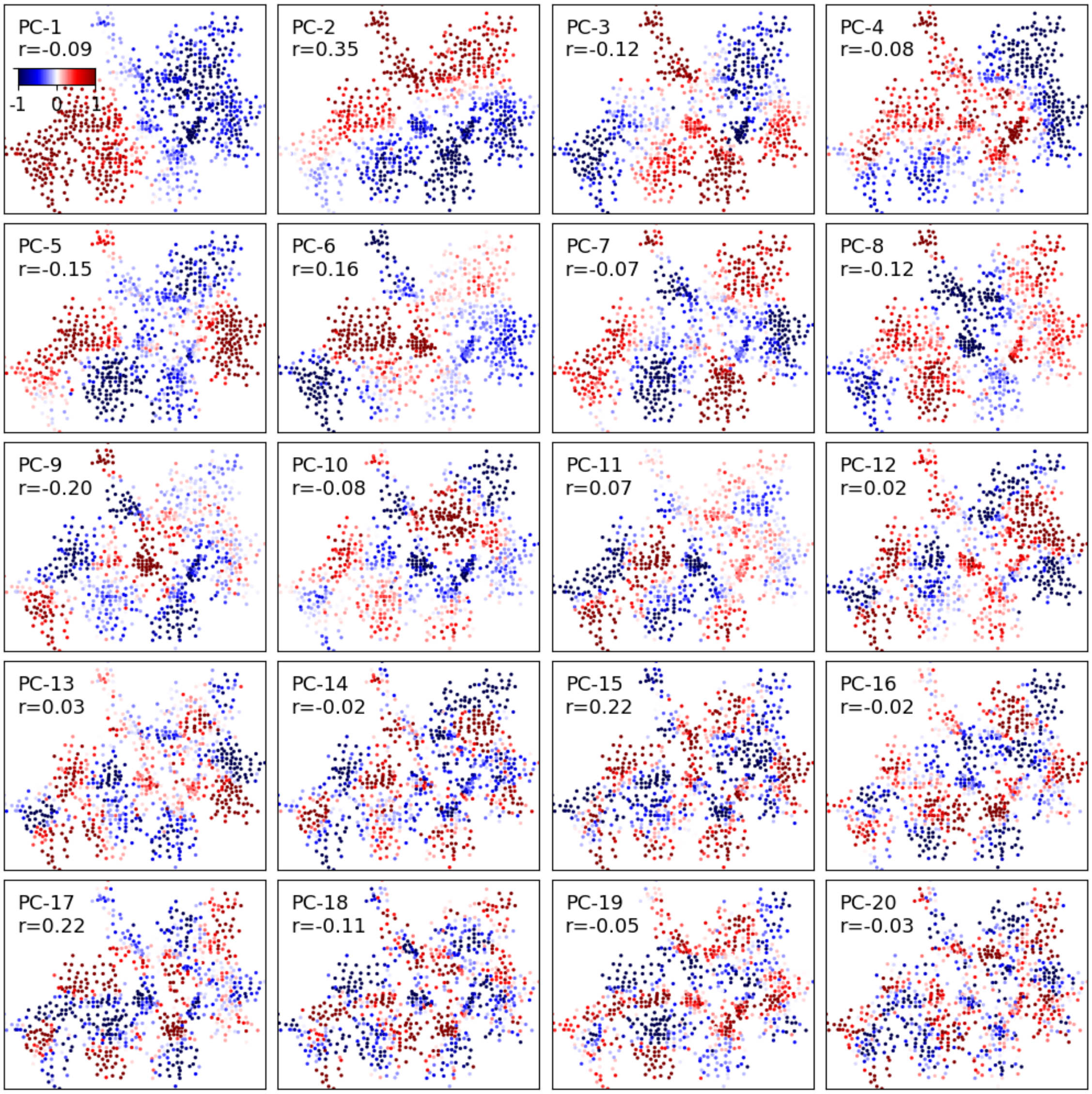

Figure S6: Spatial structure of the 20 principal components of human mobility patterns in Dallas. The correlation with neighborhood income r is shown in each panel.

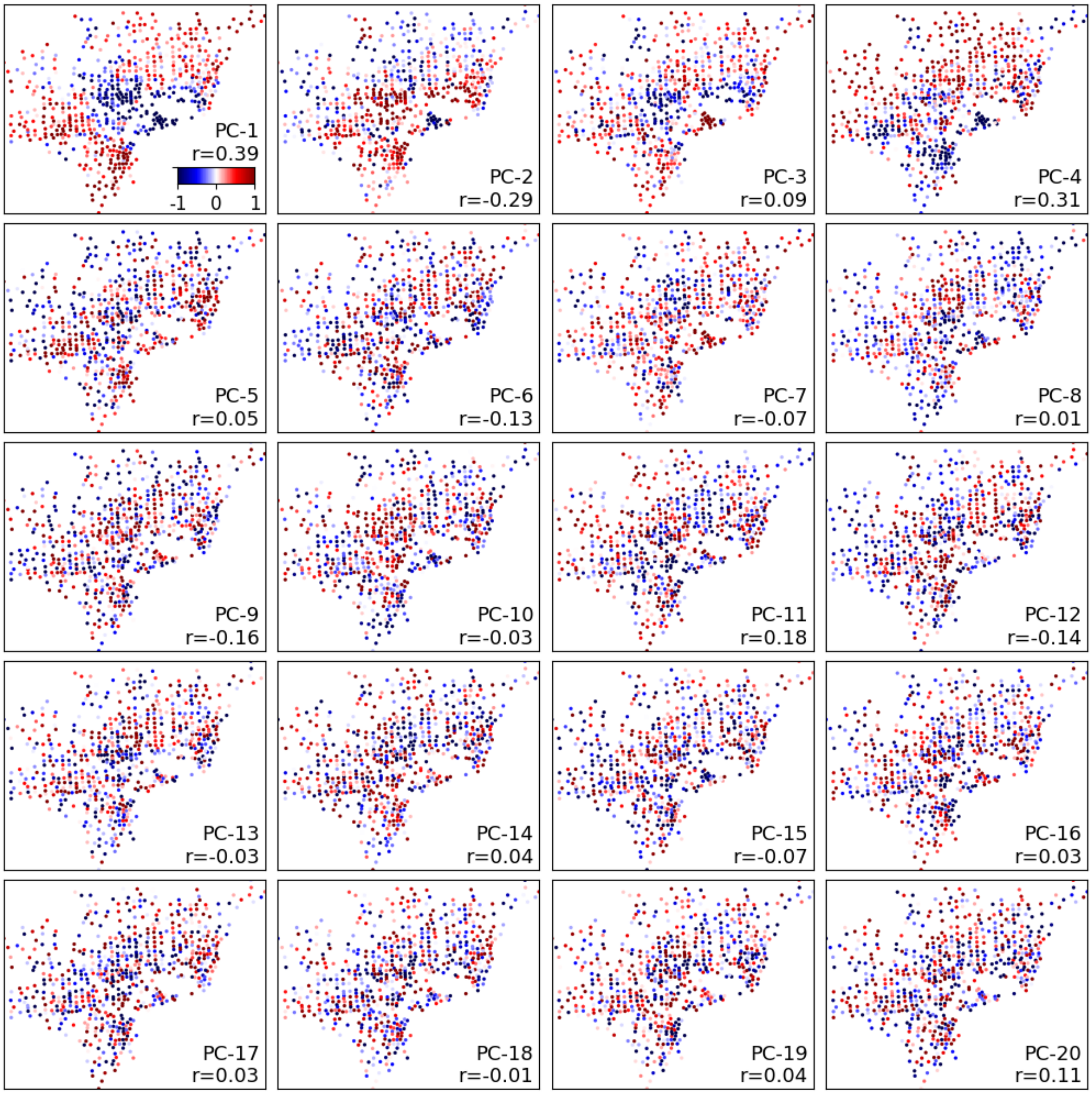

Figure S7: Spatial structure of the 20 principal components of hashtag usage in Detroit. The correlation with neighborhood income r is shown in each panel.

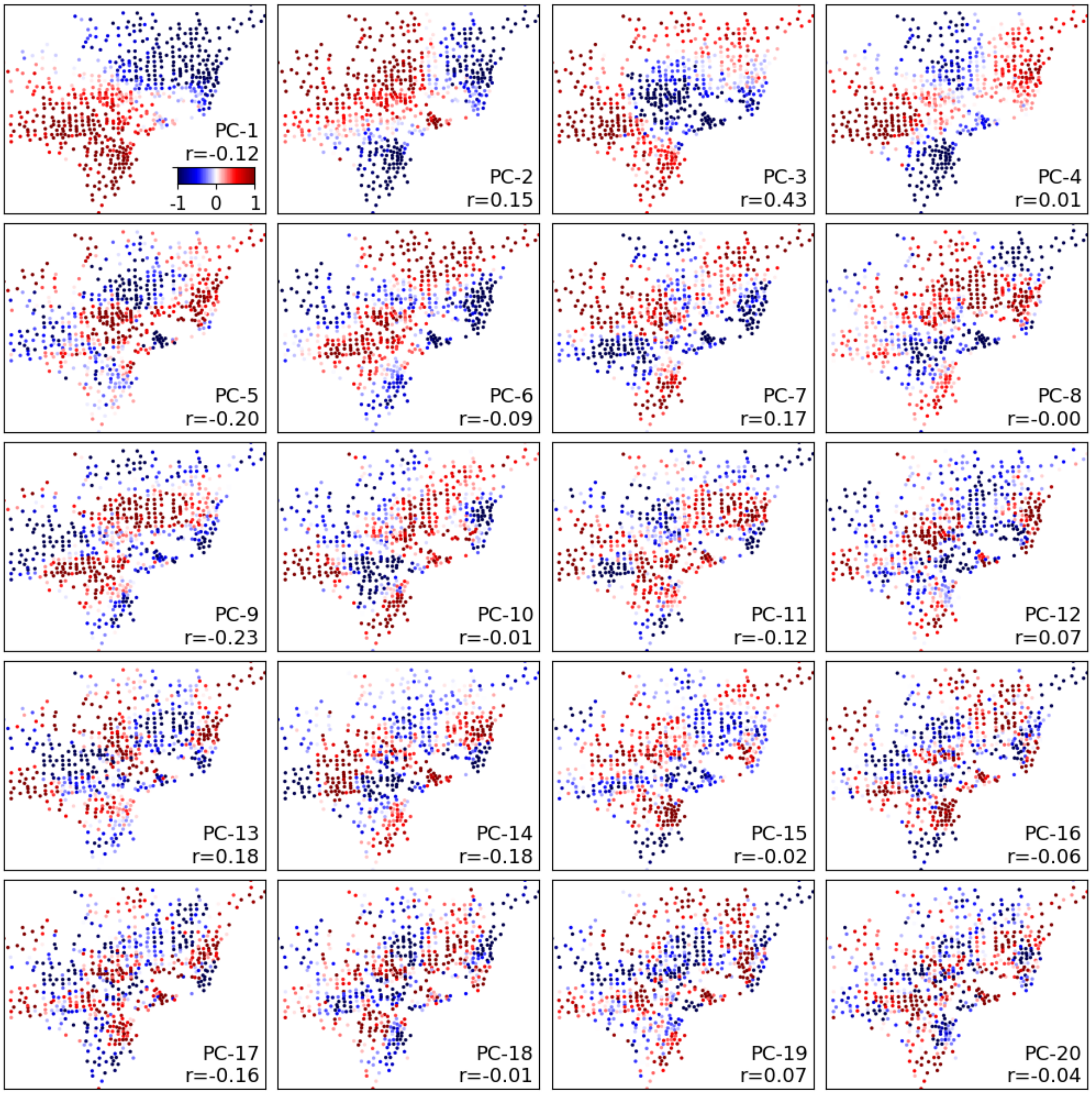

Figure S8: Spatial structure of the 20 principal components of human mobility patterns in Detroit. The correlation with neighborhood income r is shown in each panel.

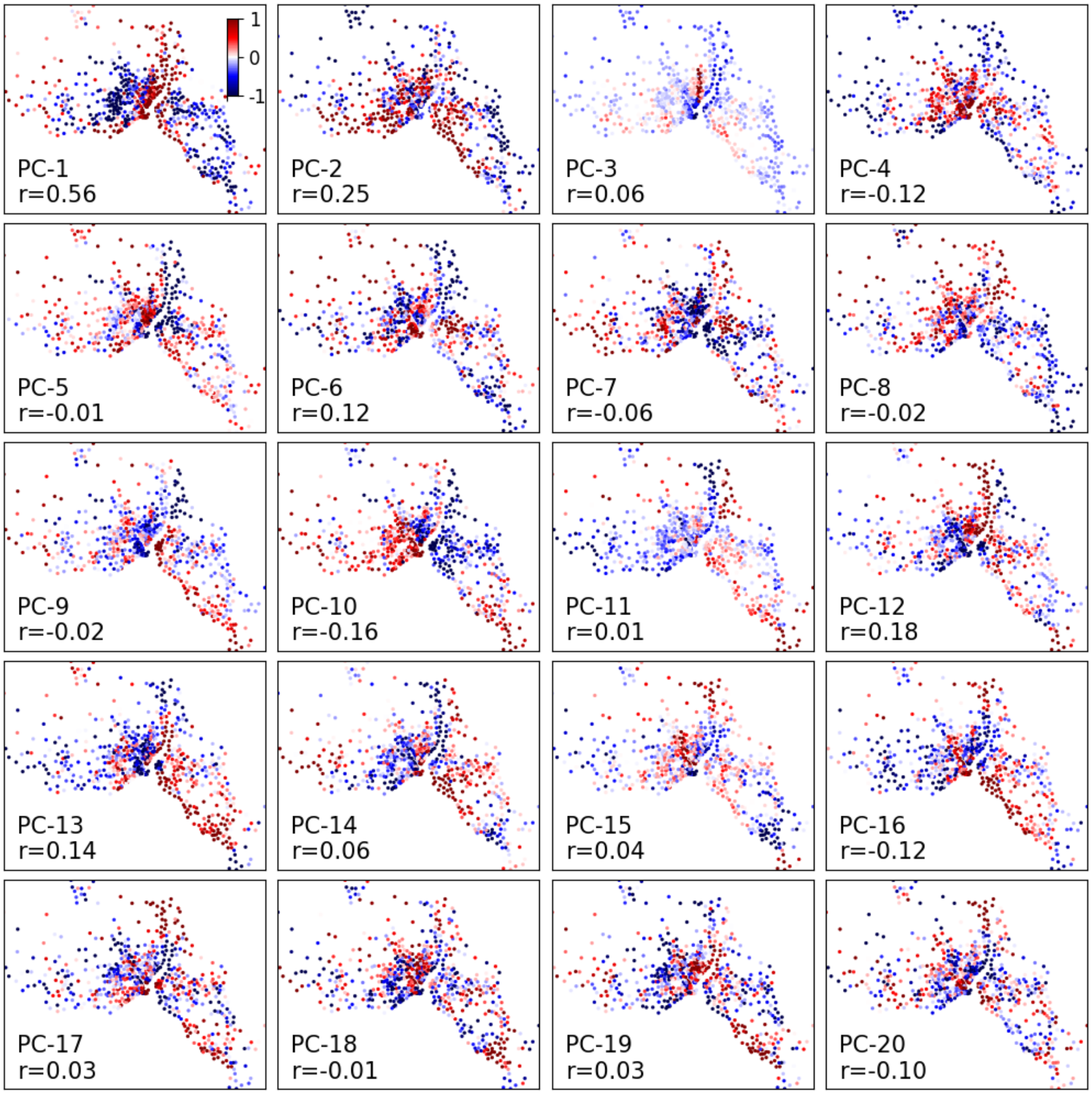

Figure S9: Spatial structure of the 20 principal components of hashtag usage in Istanbul. The correlation with neighborhood income r is shown in each panel.

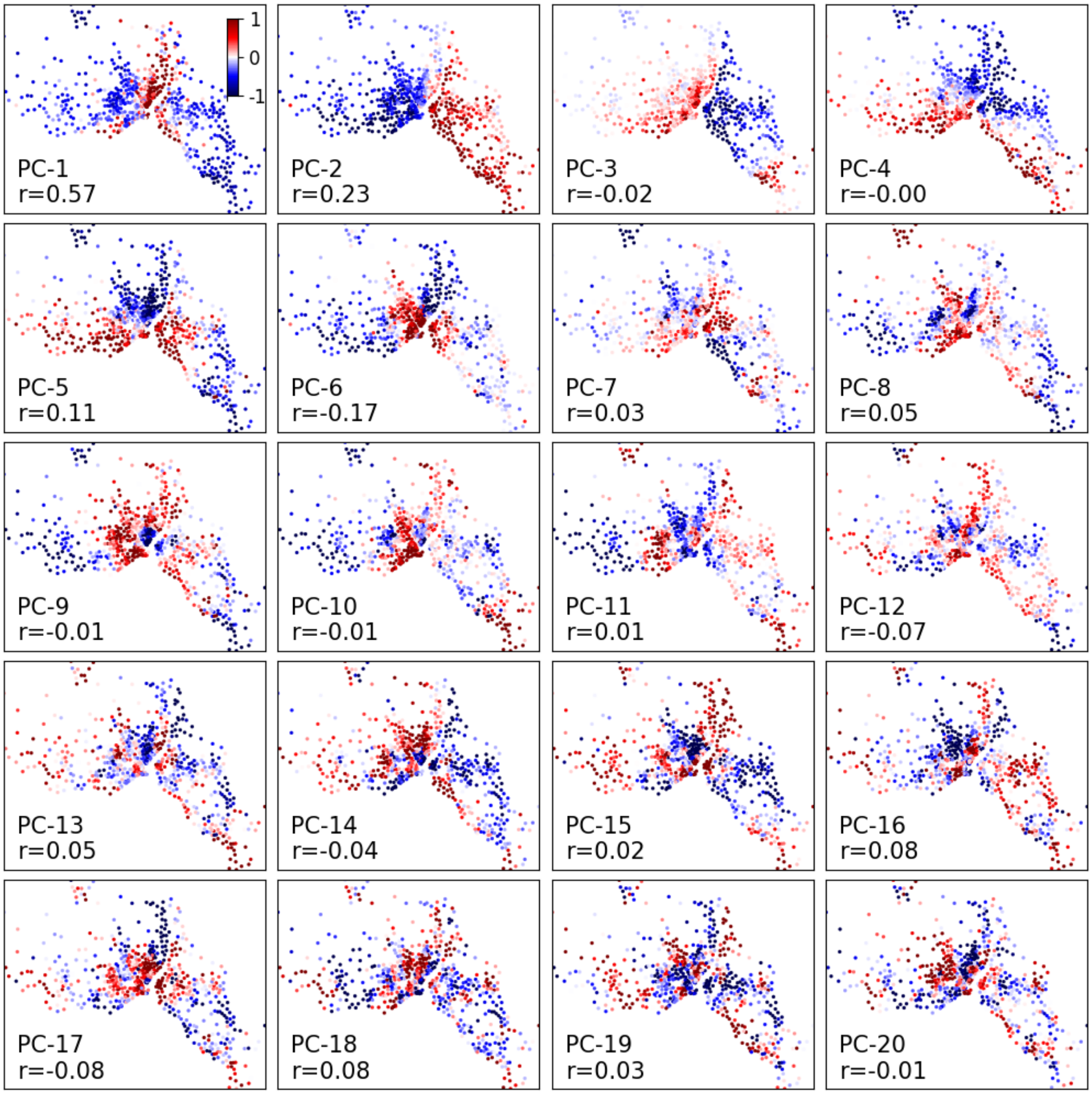

Figure S10: Spatial structure of the 20 principal components of human mobility patterns in Istanbul. The correlation with neighborhood income r is shown in each panel.

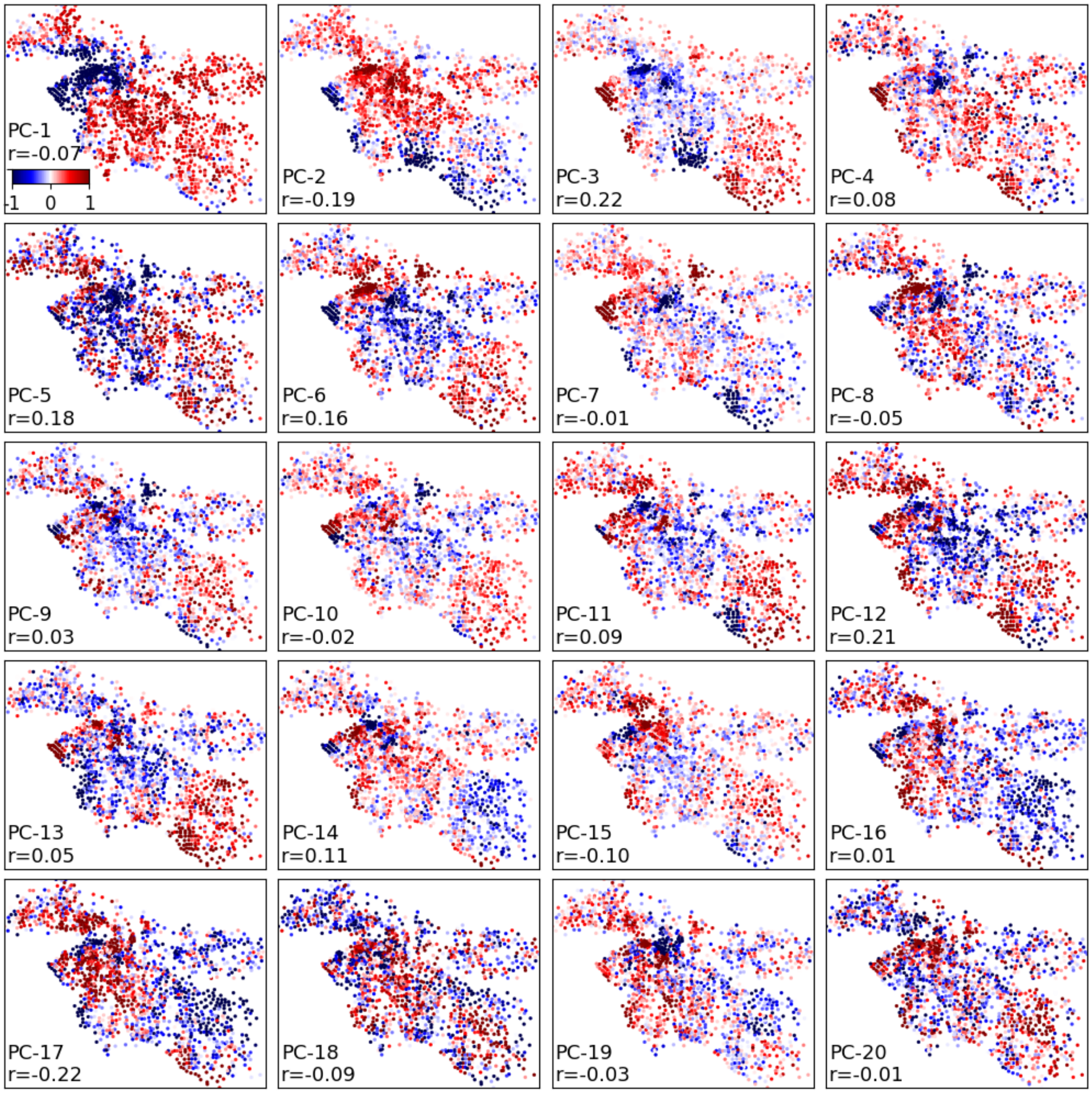

Figure S11: Spatial structure of the 20 principal components of hashtag usage in Los Angeles. The correlation with neighborhood income r is shown in each panel.

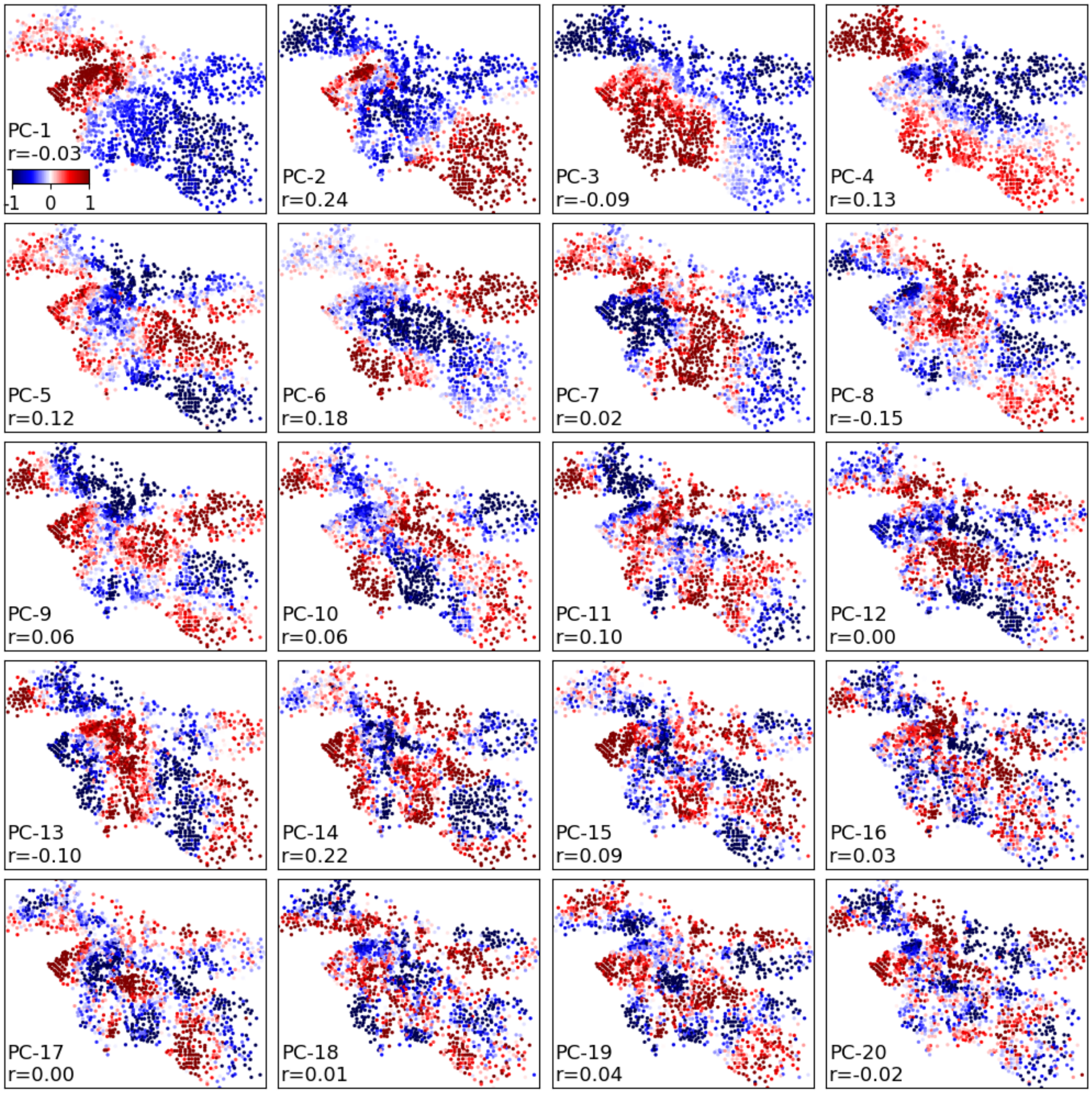

Figure S12: Spatial structure of the 20 principal components of human mobility patterns in Los Angeles. The correlation with neighborhood income r is shown in each panel.

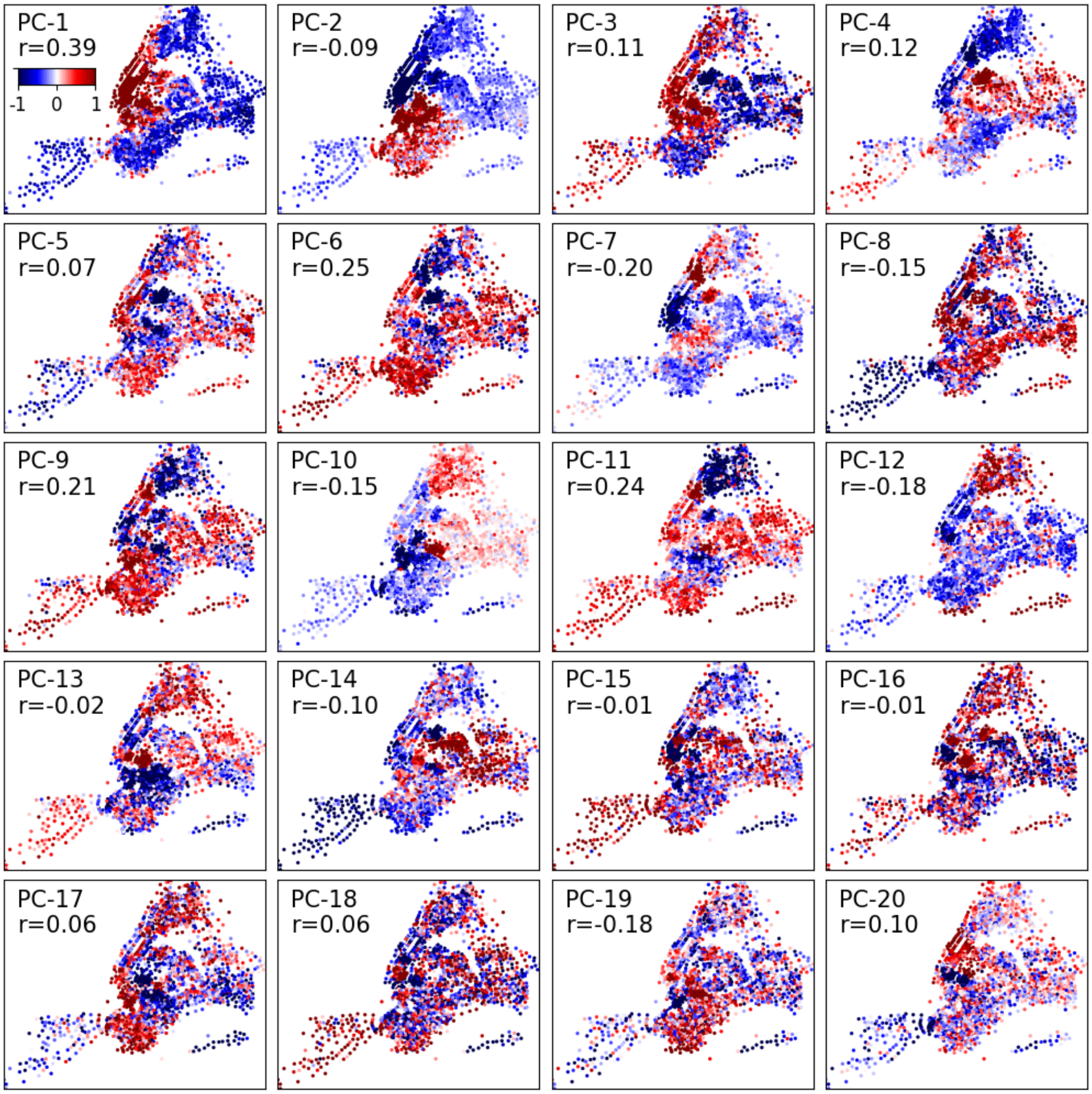

Figure S13: Spatial structure of the 20 principal components of hashtag usage in New York City. The correlation with neighborhood income r is shown in each panel.

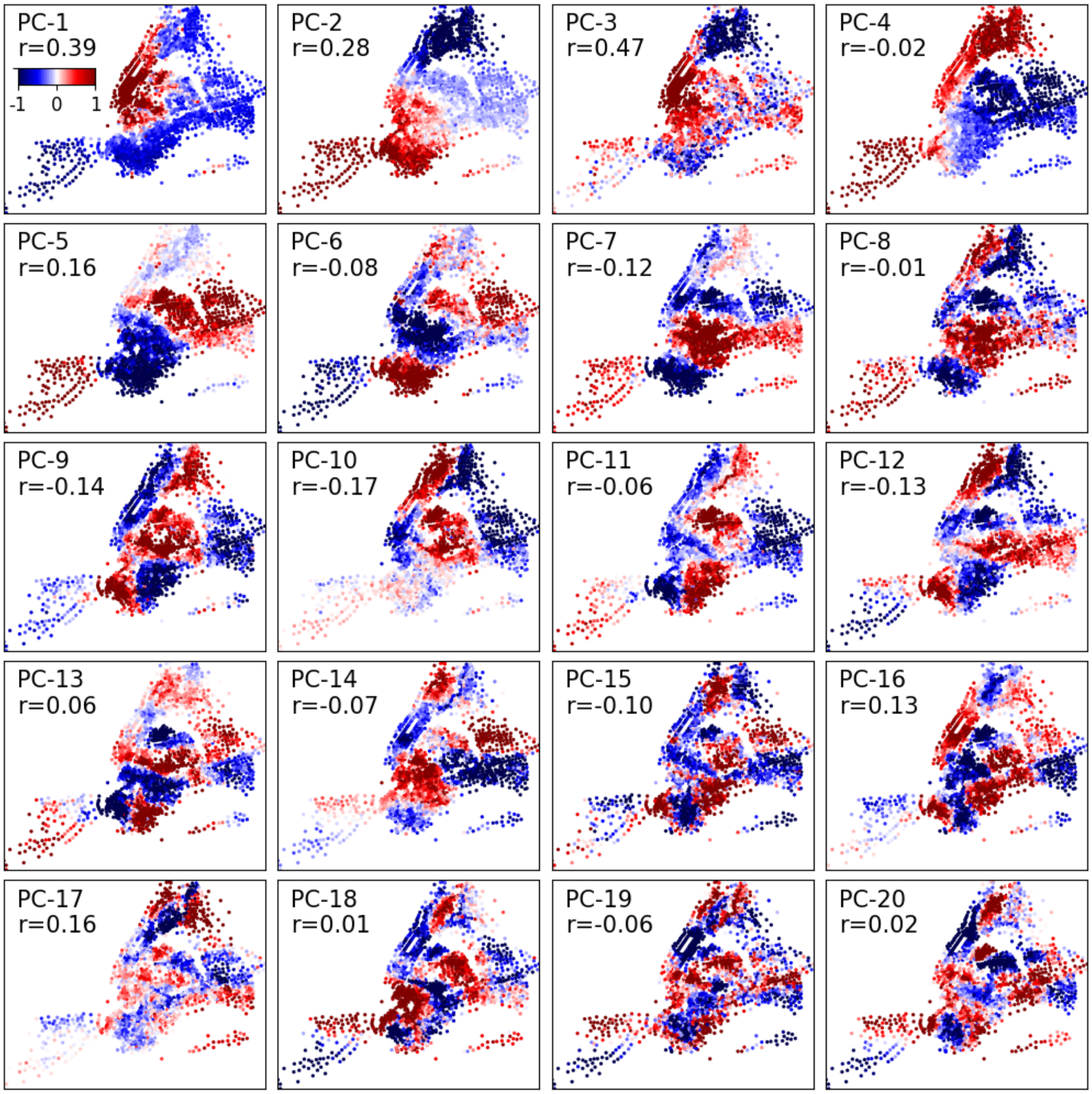

Figure S14: Spatial structure of the 20 principal components of human mobility patterns in New York City. The correlation with neighborhood income r is shown in each panel.

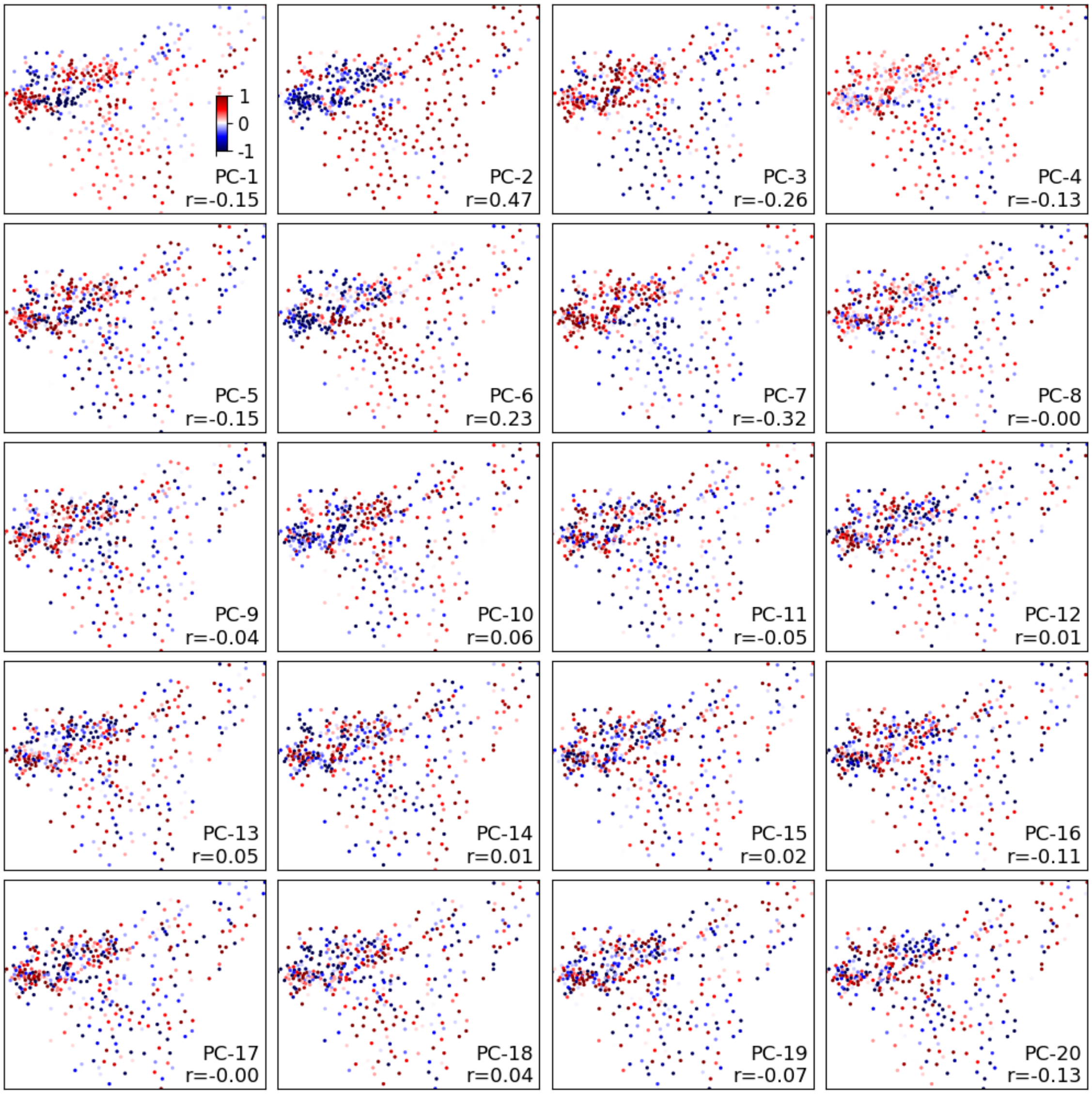

Figure S15: Spatial structure of the 20 principal components of hashtag usage in Philadelphia. The correlation with neighborhood income r is shown in each panel.

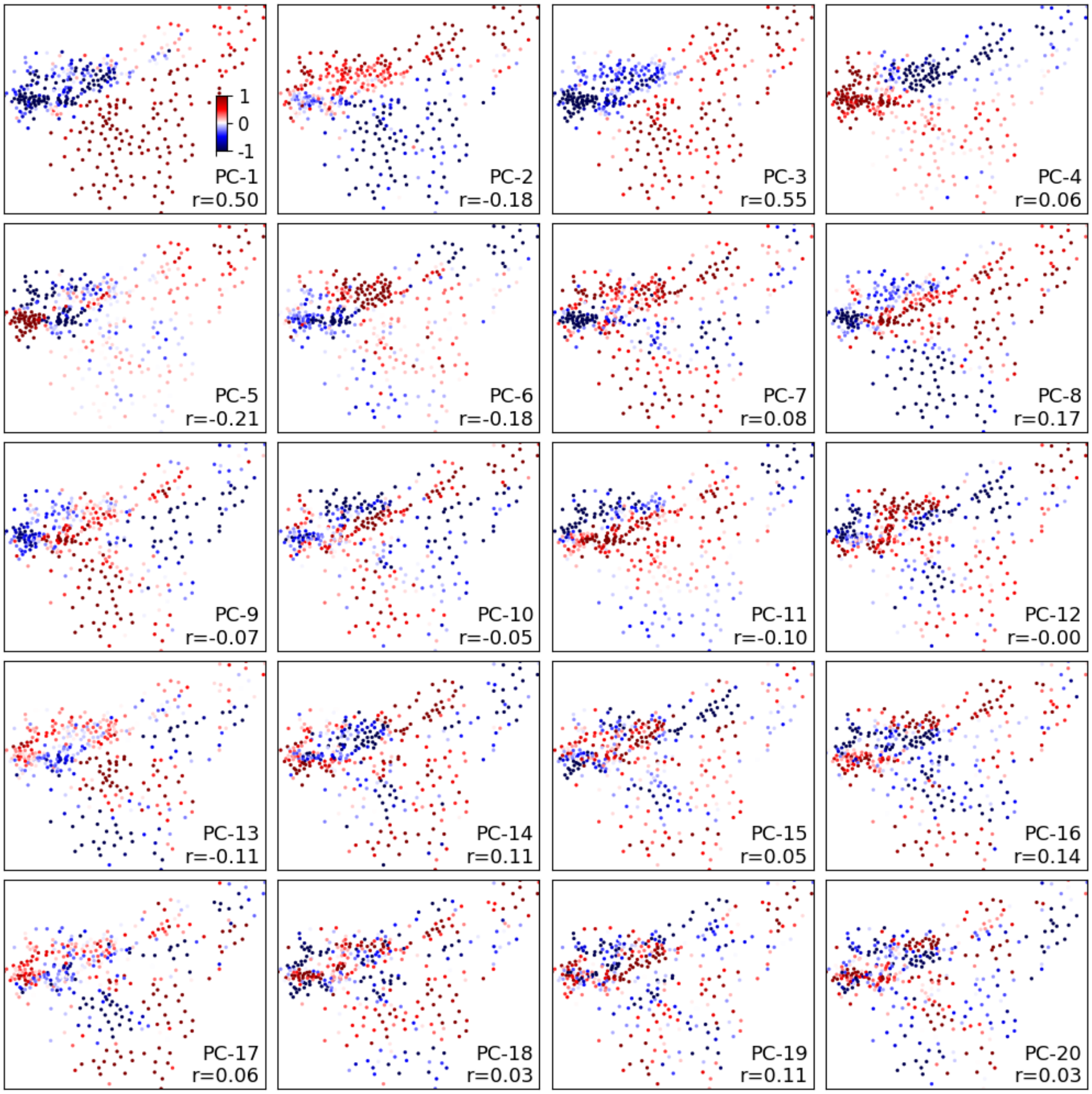

Figure S16: Spatial structure of the 20 principal components of human mobility patterns in Philadelphia. The correlation with neighborhood income r is shown in each panel.

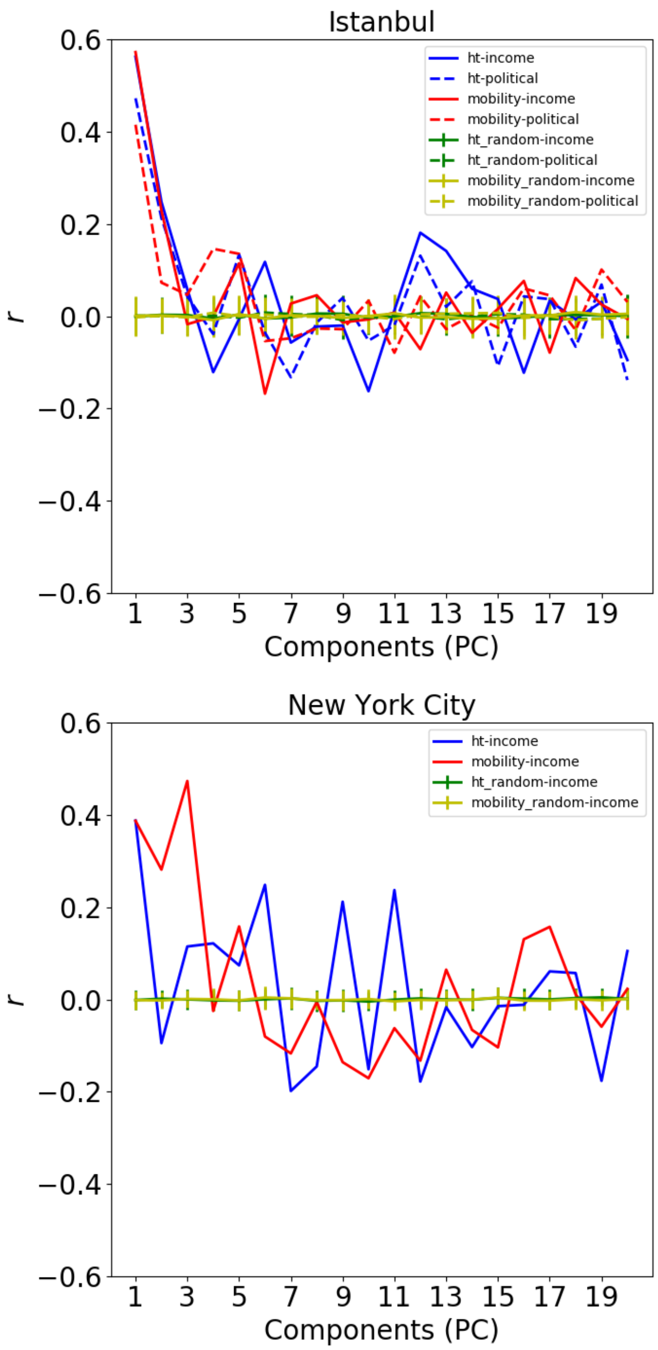

Figure S17: Correlation of the 20 principal components of hashtag usage (blue) and human mobility patterns (red) with income for Istanbul (top) and New York City (bottom). The correlation of income with the 20 principal components of a randomized hashtag (green) and mobility (yellow) space is also shown. Error bars show standard deviation. In the case of Istanbul, the correlation with electoral results is also shown (dashed lines).

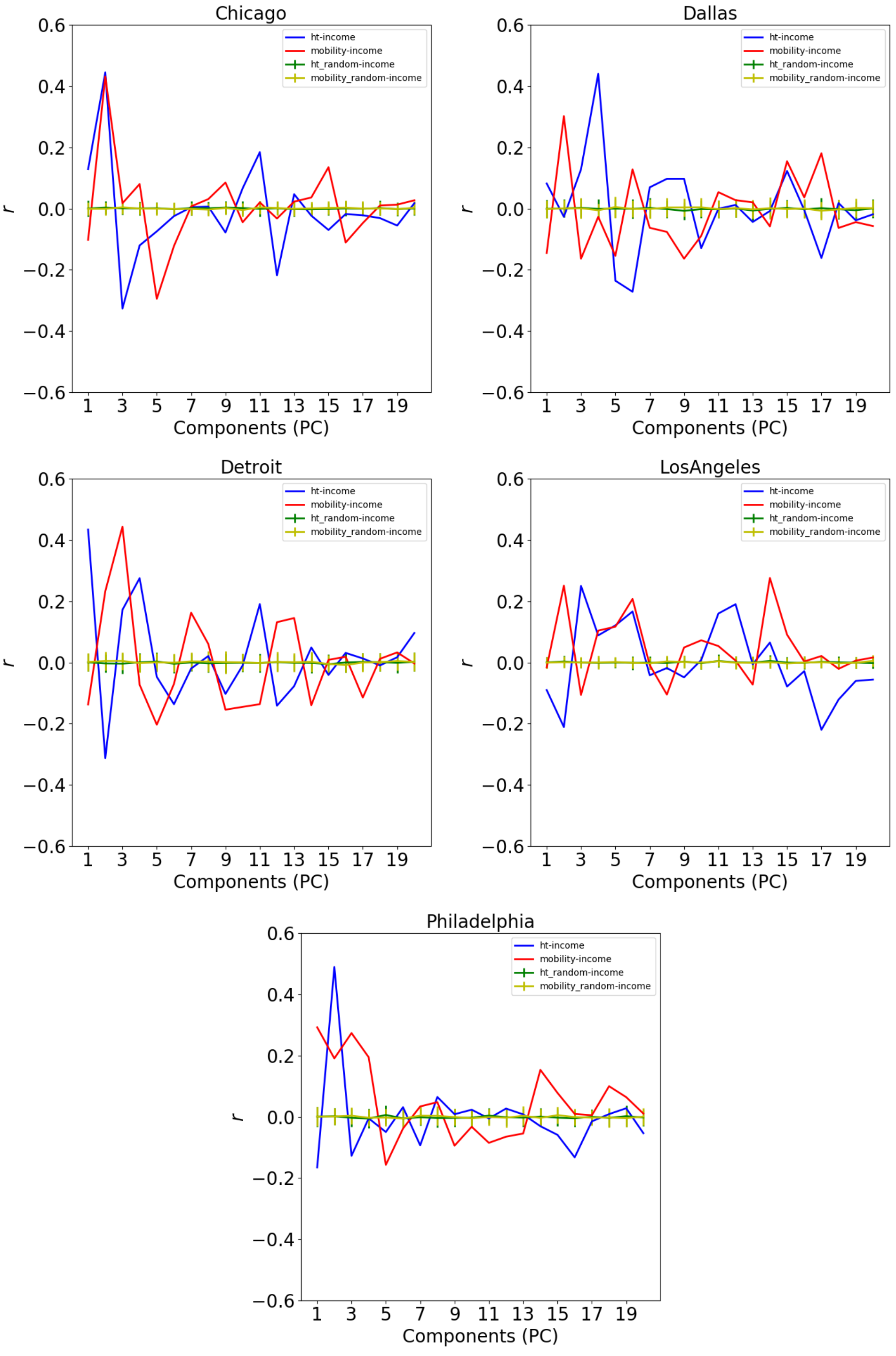

Figure S18: Correlation of the 20 principal components of hashtag usage (blue) and human mobility patterns (red) with income for multiple American cities. The correlation of income with the 20 principal components of a randomized hashtag (green) and mobility (yellow) space is also shown. Error bars show standard deviation.

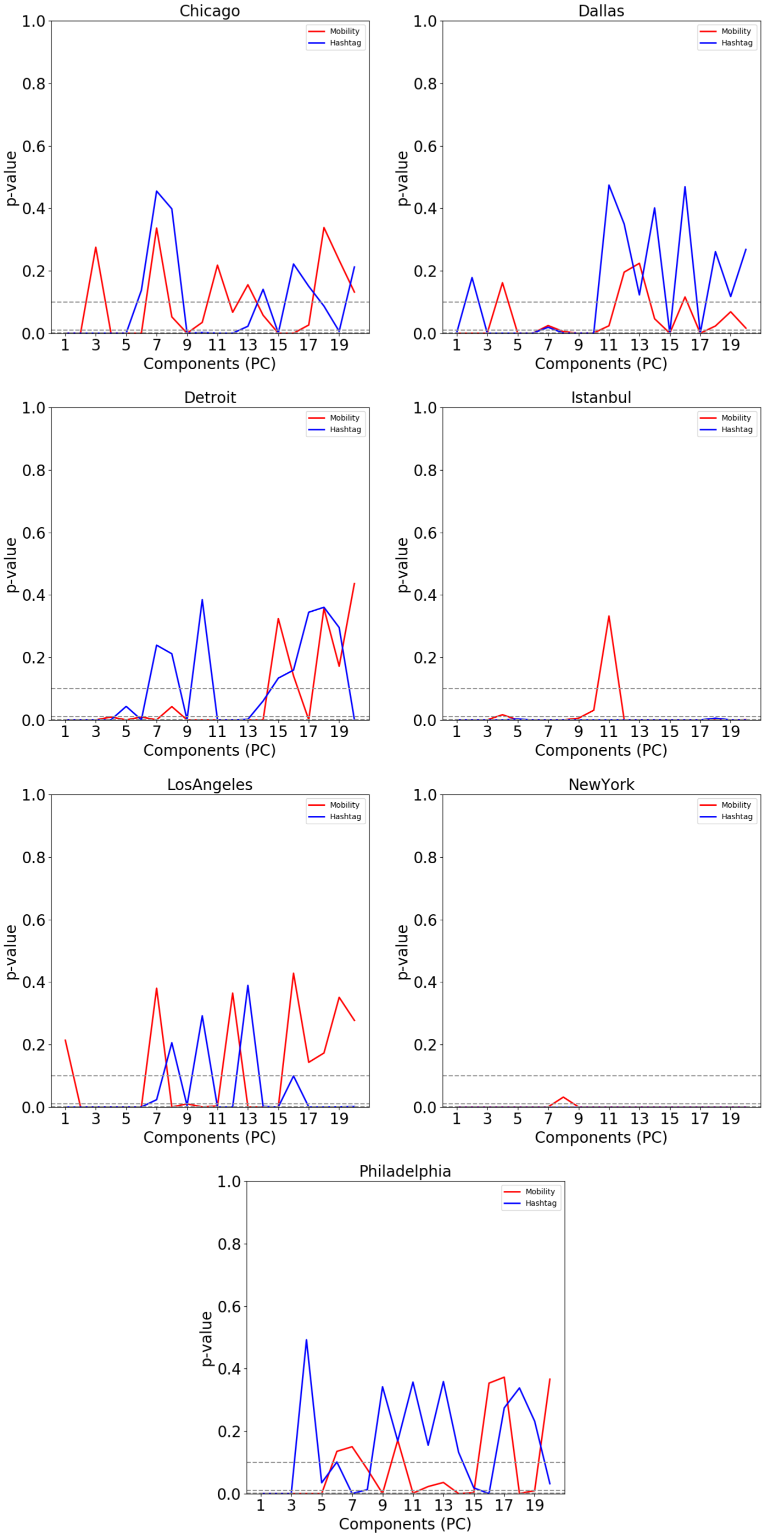

Figure S19: Probability (p-value) that the correlation of top 20 components with income results from a random distribution of hashtag (blue) and mobility (red) vectors among neighborhoods. Dashed gray lines show significance levels of 0.1, 0.01 and 0,001 from top to bottom. The correlations with income of the components (see Table 2 in the main text) shown in Figures 4 and S2 are significant ($p < 0.001$).

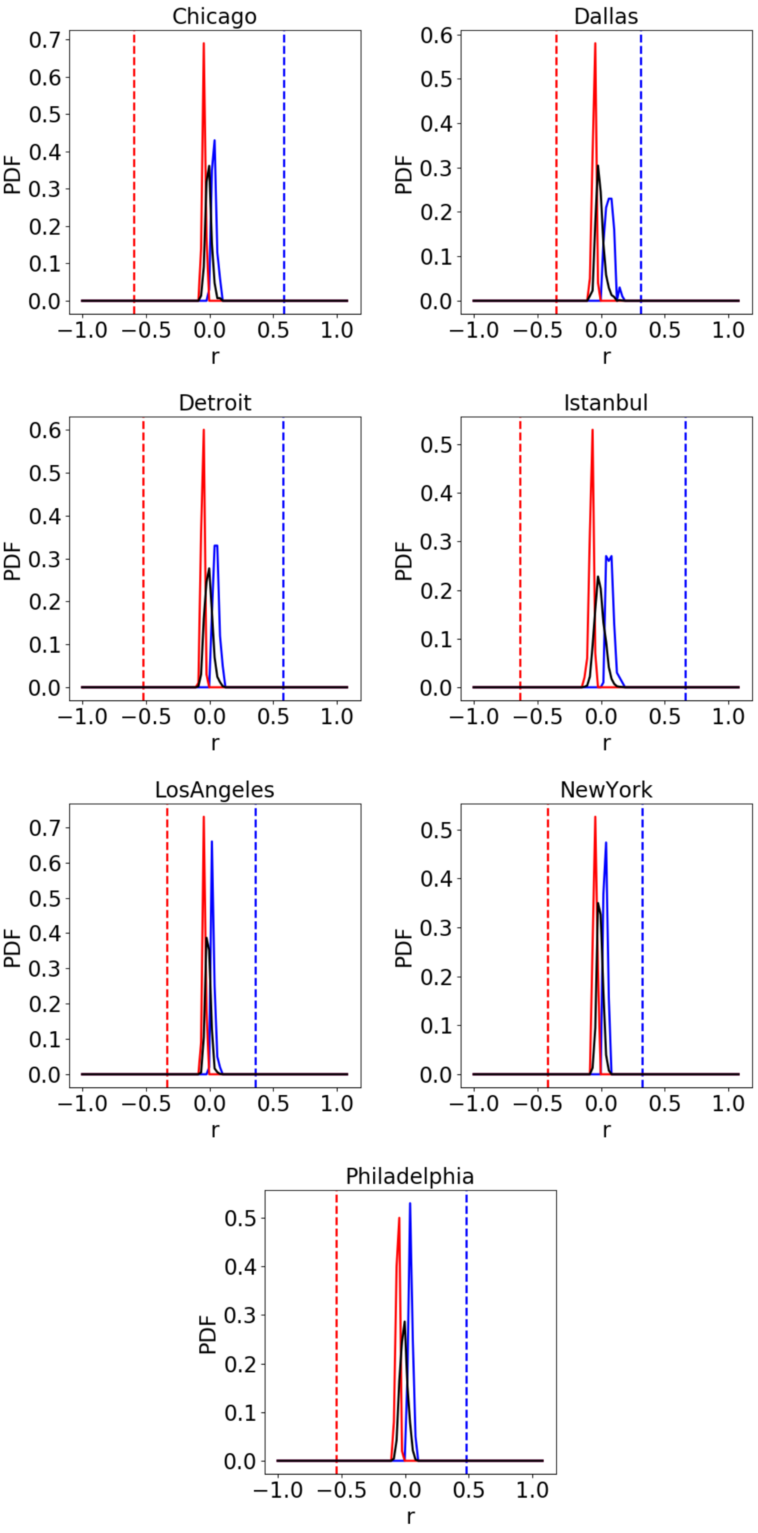

Figure S20: Correlation of hashtag topics with income. The black curve shows the distribution of correlations of income and topics from 100 randomized hashtag spaces. The blue curve shows the distribution of maximum correlation. The red curve shows the distribution of minimum correlation. The blue and red dashed lines show the actual correlations of the topics shown in Figures 5 and 6 and Table 3 from the main text.

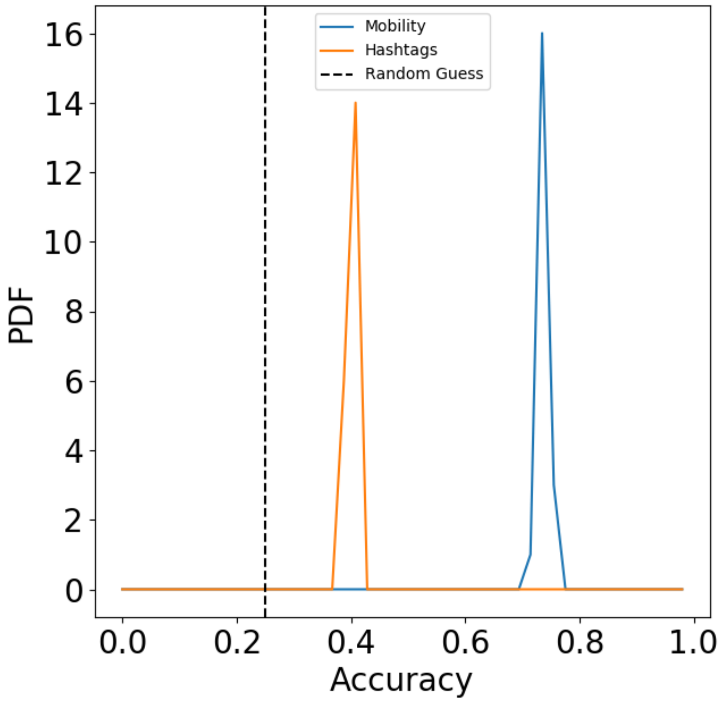

Figure S21: Accuracy of classifier predicting individual income quantile in Istanbul based on mobility (blue) and hashtag usage (orange) patterns. We show the distribution after bootstrapping the prediction and accuracy values. Colors indicate if feature space represents mobility or hashtag usage. Dashed line shows random guess.

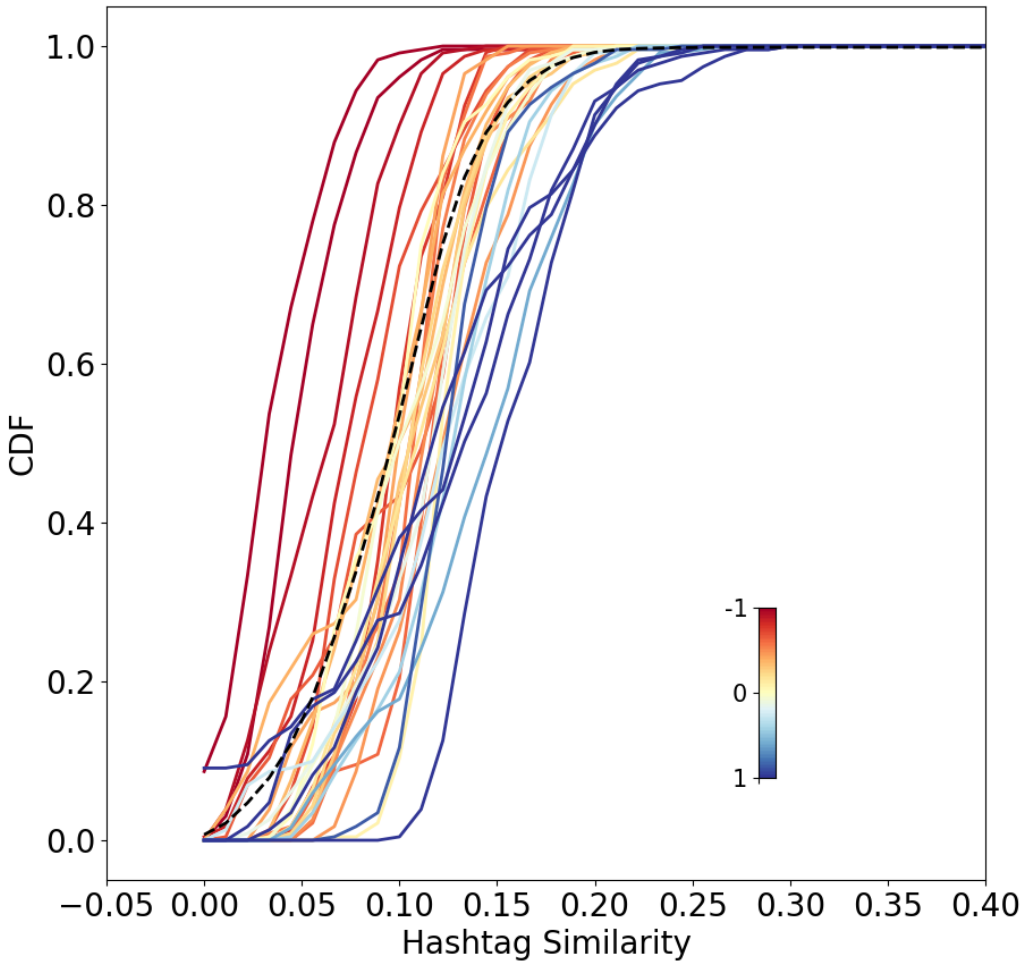

Figure S22: Cumulative density function (CDF) of pairwise neighborhood hashtag similarity. Neighborhoods are grouped by income in 30 quantiles represented with each curve. The color of each curve indicates the normalized median income of each quantile (color scale shown in figure). The black dashed curve shows the global distribution.

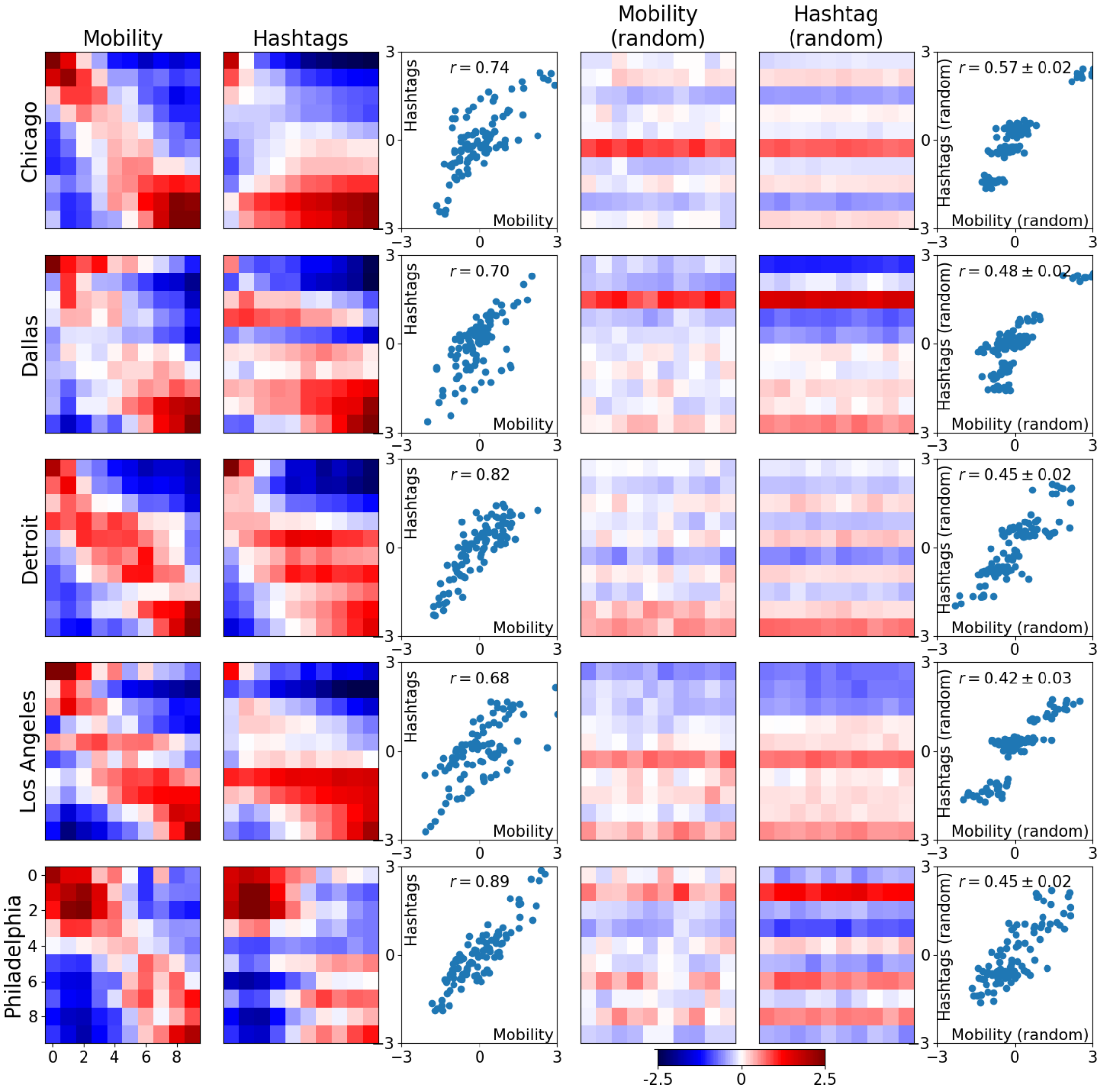

Figure S23: Inter-neighborhood social exposure through mobility and hashtag usage similarities after aggregating by income (left panels) or random association (right panels), for Chicago, Dallas, Detroit, Los Angeles and Philadelphia. Neighborhoods have been aggregated into ten income quantiles represented in the axes. Interactions depicted in blue are below the expected value from a uniform distribution and those in red above. Matrices have been normalized relative to the corresponding standard deviation from income aggregation (scale in figure). Scatter plots show the correlation (r) between mobility and hashtag similarity for both types of aggregation. In the random case (right panel), we show average correlation and standard error after 100 realizations.

City	Topic-1 ($r > 0$)	Topic-2 ($r < 0$)
Chicago	blackhawks, chicago, tbt, wcw, 1, sorrynotsorry, love, bulls, becauseitsthecup, wtf, lol, mcm, photo, bears, esurancesave30, blessed, thestruggle, truth, fml, hawks, turnup, smh, help, usa, cubs, winning, voterizzo, selfie, oops, excited, why, family, 2, gohawks, happy, yolo, beardown, targetsale, bucciovertimechallenge, cantwait, no, rip, what, ugh, dead, throwback, bye, worldcup2014, awesome, loveit, dying, coupon, finally, awkward, yes, whitesox, classic, summer, yum, fuck, illinois, lovey,chiberia, wow, letsgohawks, goodnight, oscars, not, firstworldproblems, jobs, friends, nofilter, ff, swag, tired, life, annoyed, grammys, nfl, please, fail, worldcup, college, noshame, done, job, thereturn, realtalk, mylife, oomf, tfios, neverforget, thanks, voicesave, seered, yummy, bored, snow, tgif, arg	oomf, wcw, rt, 1, mcm, turnup, np, retweet, chicago, tbt, squad, blessed, goodnight, 2, chiraq, bullsnation, love, salute, ifwedate, scandal, thiscouldbeusbutyouplayin, work, bulls, rip, goodmorning, sorrynotsorry, fact, lhhatl, fav, dead, follow, thestruggle, nationalbestfriendday, rns, smh, truth, prom2k14, gang, 100, latepost, respect, dope, selfie, aries, lmao, otf, pisces, life, bored, taurus, workflow, virgo, leo, cancer, family, scorpio, 3, wce, fml, raw, fb, yeet, facts, bears, simple, capricorn, catfish, winning, nowplaying, southside, seered, pll, throwback, period, realtalk, notalking, single, fuck, realshit, wtf, classic, 2014, soundcloud, gemini, beardown, libra, amen, nw, tired, followback, i, 5, fuckit, singlebecause, excited, like, onme, retweeet, aquarius, music

Table S7: Top hashtags from topics positively (Topic-1, $r > 0$) and negatively (Topic-2, $r < 0$) correlated with income in Chicago.

References

[1] Kalev Leetaru, Shaowen Wang, Guofeng Cao, Anand Padmanabhan, and Eric Shook. Mapping the global Twitter heartbeat: The geography of Twitter. *First Monday*, 18(5), 2013. doi: 10.5210/fm.v18i5.4366.

[2] Frank J. Massey. The Kolmogorov-Smirnov test for goodness of fit. 46(253):68–78, 1951.

[3] Jure Leskovec, Anand Rajaraman, and Jeffrey D. Ullman. *Mining of Massive Datasets*. Cambridge University Press, New York, NY, USA, 2nd edition, 2014.

[4] Marianne Bertrand and Emir Kamenica. Coming apart? Cultural distances in the United States over time. Working Paper 24771, National Bureau of Economic Research, 2018.

City	Topic-1 ($r > 0$)	Topic-2 ($r < 0$)
Dallas	ponyup, s,moodymagic, collegeslam, mustangs, nit, sportsbiz, 6thfan, newmoody, theworkisworthit, tfm, smubasketball, moodymadness, familyweekend, payne10, l,beone, smufootball, finishtherightway, hpumc, boulevarding, leadercastdallas, smuhomecoming, homecoming, be1, ponyexpress, 43, whatismylife, leadercast, wrecktech, l1c4, collegebound, nbd, mustang, finalsweekgotmelike, b1, plaid, gomustangs, timelapse, itscasual, maytheoddsbeeverinyourfavor, triblive, heels, dubya, whatissleep, commitment, 2days, swagger, anxiety, finalsweek, sorryboutit, ucf, badtiming, chikfila, alwaysandforever, biglittle, irock, cantwalk, nocomplaints, godfather, footballgame, we, sarah, cookiemonster, comefaster, hellweek, treats, sistasista, ringbyspring, archer, getitdone, favoritethings, dgaf, cbb, cal, thanksmommy, halo, reasonstolive, kappa, whiteboyproblems, 3years, playhard, kidatheart, goodstart, scarredforlife, multicultural, dallas, lic, jungle, getit, utfollowtrain, upset, tbt, beatuconn, butforreal, tylerseguin, scifiexpo, college, crazydoglady, strugglin	vegasbaby, gaga, concert, xtc, whoknows, belieber, raptor, witchcraft, legends, lahhatl, goodfriend, gobigorgohome, jam, turnip, realestate, science, selfieolympics, believe, oomf, gocowboys, sbsw, tbt, truth, wcw, turnup, dallas, 1, raw, truestory, fml, thestruggle, lonestarpark, mexico, usa, wreckem, cowboysnation, blessed, nssc10, mcm, cowboys, ouch, smart2014, selfie, thiscouldbeusbutyouplayin, esurancesave30, texas, ss, love, worldcup2014, dead, classic, cincodemayo, lonestarselfie, tgif, ger, mffl, goodnight, boomer, lifeisgood, 2, oops, gameofthrones, thanks, mavs, sorrynotsorry, usmnt, honestyhour, humpday, happy, work, tonight, kidcudi, arg, nosleep, christmas, mtvstars, coupon, kentuckyderby, dallasweather, gohawks, prom2k14, sec, twitter, swag, goodfriday, raceforthecure, art, nationalbestfriendday, cancer, leadandsucceed, help, fran, ready, merica, nbafinals, vampireweekend, gostros, pisces, 24lad

Table S8: Top hashtags from topics positively (Topic-1, $r > 0$) and negatively (Topic-2, $r < 0$) correlated with income in Dallas.

City	Topic-1 ($r > 0$)	Topic-2 ($r < 0$)
Detroit	wcw, mcm, tbt, oomf, 1, goblue, sorrynotsorry, turnup, love, michigan, esurancesave30, thiscouldbeusbutyouplayin, truth, tigers, fml, thestruggle, wtf, 2, lol, rip, blessed, smh, ifwedate, np, throwback, oops, tb, dead, bucciovertimechallenge, selfie, goodnight, cantwait, why, puremichigan, bored, usa, tweetabond, yolo, nationalbestfriendday, what, family, help, winning, happy, subtweet, excited, lions, pll, pickrick, awkward, life, redwings, please, fuck, noshame, not, gogreen, no, tired, voicesave, awesome, bootyhadmelike, classic, loveher, finally, swag, wow, yes, gowings, thewalkingdead, loveit, ugh, ohwell, respect, bye, throwbackthursday, dying, ff, thefamily, webelievedet, work, tfios, neverforget, annoyed, perfect, m,ouch, done, 3, fact, singlebecause, mylife, damn, thanky,letsgo, turndownforwhat, perksofdatingme, exhausted, lovey,pissed	oomf, detroit, retweet, rt, bible, period, lhhatl, scandal, wcw, fav, 1, tbt, goodmorning, salute, np, goodnight, 2, retweeet, starlife, pisces, oomfs, turnup, girlfriendtweet, virgo, dead, classic, single, birthdaytweet, nf, fb, cancer, taurus, simple, michigan, wce, bbwla, fact, smh, blessed, gemini, mcm, nowplaying, free, havesandhavenots, baetweet, aries, followback, justwaitonit, follow, dope, monumentour, prom2k14, subtweet, support, rhoa, love, staycrispy, swear, letitrip, work, nofilter, now, 2014, leo, favorite, workflow, lrt, raw, shoutout, lhhny, bandgang, soundcloud, faith, boyfriendtweet, dte, stunthard, aquarius, repost, teamfollowback, yeet, thenine, damn, god, scorpio, libra, 300, thasummerblast, rip, virgos, listen, truth, s, happymothersday, pontiac, loveinthacity, followed, 100, freebandz, realtalk, loyalty

Table S9: Top hashtags from topics positively (Topic-1, $r > 0$) and negatively (Topic-2, $r < 0$) correlated with income in Detroit.

City	Topic-1 ($r > 0$)	Topic-2 ($r < 0$)
Los Angeles	tbt, wcw, california, 1, love, blessed, lax, mcm, sorrynotsorry, usa, family, dodgers, lol, selfie, nofilter, fml, socal, thestruggle, 2, truth, la, np, gokingsgo, sunset, wtf, smh, earthquake, life, cali, angels, happy, summer, winning, ucla, turnup, fitness, goodnight, starbucks, sundayfunday, photo, beautiful, yolo, beach, arg, losangeles, dead, fun, worldcup, oomf, oc, worldcup2014, vegas, rip, hiking, newportbeach, esurancesave30, santamonica, tgif, oops, food, firstworldproblems, friends, throwback, ,travel, help, foodporn, why, home, mothersday, 3, swag, nature, orangecounty, goodtimes, workout, ger, thiscouldbeusbutyouplayin, work, college, lakings, lagunabeach, awkward, vscocam, realtalk, respect, awesome, gkg, bye, excited, justsaying, springbreak, no, goodmorning, halloween, subtweet, tb, hike, fail, tired	wcw, oomf, mcm, disney, sorrynotsorry, np, 1, ifwedate, ,nationalbestfriendday, nw, anaheim, thestruggle, thiscouldbeusbutyouplayin, tb, sadtweet, tbt, goodnight, quotethiswithapictureofus, noerapenal, bww, dead, 2, wondercon, fml, thestruggleisreal, ger, pll, dca, smh, subtweet, turnup, votegrich, liestoldontwitter, relationshipgoals, leo, pisces, tfios, bomb, done, californiaadventure, wce, foreveralone, youremoreattractiveif, splashmountain, js, starbucks, singlebecause, scorpio, excited, expowest, knottsberryfarm, memories, captainamerica, mickeymouse, classof2014, uci, thewalkingdead, selfie, disneyside, teamcanelo, sealbeach, icant, lakers, hauntedmansion, wondercon2014, bored, citrixsynergy, eij13, wtf, teamnosleep, aries, family, justsaying, happiestplaceonearth, tweetabondthatcantbebroken, judgeme, marvel, libra, stopmexicangirls2014, wecantdateif, priceless, 10, bethanymotagiveaway, photo, stopmexicangirls2013, annoyed, tweetyourweakness, usa, knotts, downtowndisney, nowplaying, fail, lakersnation, fake, skatepark4logan, buffalowildwings, rip, halamadrid

Table S10: Top hashtags from topics positively (Topic-1, $r > 0$) and negatively (Topic-2, $r < 0$) correlated with income in Los Angeles.

City	Topic-1 ($r > 0$)	Topic-2 ($r < 0$)
New York City	nyc, NYC, newyork, timessquare, manhattan, NewYork, newyorkcity, ny, love, TimesSquare, NY, broadway, usa, tbt, timesquare, latergram, nofilter, selfie, Manhattan, nyfw, summer, fashion, art, friends, vscocam, travel, NewYorkCity, USA, foodporn, fun, Broadway, food, NYFW, city, happy, amazing, brunch, family, beautiful, sunset, instagood, music, soho, yum, me, empirestatebuilding, night, architecture, subway, dinner, bigapple, photo, TimeSquare, birthday, rooftop, spring, empirestate, shakeshack, worldcup, SuperBowl, foodie, 100happydays, regram, coffee, iloveny, party, lunch, style, superbowl, vsco, photooftheday, lights, work, midtown, streetart, WorldCup, vacation, TBT, skyline, awesome, beauty, blessed, view, ootd, chelsea, highline, freedomtower, life, WorldCup2014, photography, america, tgif, goodtimes, design, starbucks, eastvillage, yummy, mbfw, friday, sundayfunday	oomf, yankees, Yankees, wcw, bronx, facts, tbt, WCW, Bronx, mcm, RT, NBLNabilaVoto, replytweet, yankeestadium, Facts, Oomf, TBT, Job, baseball, sorrynotsorry, YankeeStadium, IfWeDate, selfie, tb, Jobs, LHHATL, turnup, ThisCouldBeUsButYouPlayin, MCM, Pisces, jerseycity, np, JerseyCity, NYY, Gemini, goodnight, BRA, Leo, RE2PECT, BETAwards, Sagittarius, COL, Scorpio, Virgo, FACTS, TweetMyJobs, Aries, family, TurnUp, PLL, TeenWolf, Taurus, Libra, Cancer, Capricorn, NP, GoodMorning, USA, love, RelationshipGoals, Scandal, thestruggle, RNS, RAW, ARG, MTVHottest, GER, TheWalkingDead, wce, RIP, BETAwards2014, Blessed, Aquarius, VMAs, LRT, PremiosJuventud, nyy, life, CallMeCam, Tbt, honestyhour, smh, HeatNation, SoundCloud, OITNB, TGIF, respect, HonestyHour, WorldCup2014, throwback, oomfs, WWE, Family, jeter, E,Retweet, Salute, Np, Jeter, rt

Table S11: Top hashtags from topics positively (Topic-1, $r > 0$) and negatively (Topic-2, $r < 0$) correlated with income in New York City.

City	Topic-1 ($r > 0$)	Topic-2 ($r < 0$)
Philadelphia	oomf, tbt, wcw, 1, jobs, sorrynotsorry, mcm, thestruggle, blessed, replytweet, job, turnup, eagles, esurancesave30, help, wtf, 2, truth, fml, birdgang, lol, voicesave, rip, love, tweetmyjobs, bucciovertimechallenge, goodnight, neverforget, dead, coupon, why, throwback, selfie, grammys, oops, cantwait, usa, smh, pll, yolo, classic, firstworldproblems, justsaying, ugh, flyers, family, subtweet, flyeaglesfly, longislandmedium, fuck, np, thiscouldbeusbutyouplayin, tfios, bored, thewalkingdead, winning, pissed, obsessed, fact, please, awkward, mylife, wow, fail, ouch, facts, what, thanks, work, excited, thanky,ahscoven, sales, college, life, finally, success, worldcup2014, bye, gameofthrones, clutch, rt, no, workflow, retweet, ahs, ifwedate, becauseitsthecup, turndownforwhat, nursing, letsgo, bootyhadmelike, tired, respect, merrychristmas, cancer, honestyhour, addicted, motivation, christmas	ttm, oomf, rt, doop, fav, lhhatl, wcw, 1omf, stadiumtweets, justwaitonit, union, ha, soccer, oomfs, lrt, blocked, confessionhour, 1, notupfordebate, salute, retweeet, np, hoesbelike, retweet, philadelphiaunion, pplpark, tb, 2k14, pisces, rno, simple, realshit, squad, knowdat, sesly, mls, lv, leo, accmlax, chester, septa, ct,otf, sagittarius, yeet, moneyteam, heatnation, idc, freemydad, fuckit, teamheat, cancer, real, that, eagles, dc3, collegecup, beyondsoccer, respect, tityfollowtrain, worldcup, thebrothers, 400, crmc, connections, hannnn, talkheavy, greecevsnigeria, 1omfs, thebid, hdm, winning, nigeria, always, workoutflow, familyfirst, d,cashout, greece, restwell, pip, warready, ftb, sadbuttrue, soultape3, roadtobrazil, goodmusic, acc, scandal, pennsylvania, girlfriendtweet, rip, ppl, blessed, trill, fwm, photo, r, textgram, hedontbangwithy

Table S12: Top hashtags from topics positively (Topic-1, $r > 0$) and negatively (Topic-2, $r < 0$) correlated with income in Philadelphia.
